# Accelerometer-measured physical activity, sedentary behavior, and risk of incident pelvic organ prolapse: a prospective cohort study in the UK Biobank

**DOI:** 10.1186/s12966-024-01559-w

**Published:** 2024-02-02

**Authors:** Keyi Si, Zhi Cao, Qianqian Liu, Yingying Yang, Qingqiang Dai, Yuting Yao, Yingying Qiao, Chenjie Xu, Guizhu Wu

**Affiliations:** 1 Department of Gynecology, Shanghai Key Laboratory of Maternal Fetal Medicine, Shanghai Institute of Maternal-Fetal Medicine and Gynecologic Oncology, Shanghai First Maternity and Infant Hospital, School of Medicine, Tongji University, No.2699 West Gaoke Road, Shanghai, 201204 China; 2 School of Public Health, Hangzhou Normal University, No.2318 Yuhangtang Road, Yuhang District, Hangzhou, 311121 China; 3 School of Public Health, Zhejiang University School of Medicine, Hangzhou, China; 4 Clinical Research Center, Shanghai Key Laboratory of Maternal Fetal Medicine, Shanghai Institute of Maternal-Fetal Medicine and Gynecologic Oncology, Shanghai First Maternity and Infant Hospital, School of Medicine, Tongji University, Shanghai, China

**Keywords:** Accelerometer, Physical activity, Sedentary behavior, Pelvic organ prolapse

## Abstract

**Background:**

Previous studies on physical activity (PA) and pelvic organ prolapse (POP) were largely limited to self-reported PA in athletes, soldiers, and women in postpartum. We aimed to investigate the association of accelerometer-measured PA and sedentary behavior with the risk of POP in middle-aged and elderly women.

**Methods:**

In this prospective cohort derived from the UK Biobank, the intensity and duration of PA and sedentary behavior were measured with wrist-worn accelerometers over a 7-day period in 2013–2015 for 47,674 participants (aged 42.8–77.9 years) without pre-existing POP. Participants were followed up until the end of 2022, during which incident POP was ascertained mainly by the electronic health records. Multivariable-adjusted Cox proportional hazards models and restricted cubic splines were used to assess the associations of interest. Isotemporal substitution models were applied to test the effects of substituting a type of activity with equivalent duration of others.

**Results:**

During a median follow-up of 8.0 years, 779 cases of POP were recorded. The duration of light-intensity PA (LPA) was positively whereas sedentary time was negatively associated with the risk of POP. Every additional 1 h/day of LPA elevated the risk of POP by 18% (95% confidence interval [CI], 10%–26%). In contrast, the risk decreased by 5% (95% CI, 0–8%) per 1 h/day increment in sedentary behavior. No associations were found between moderate-intensity PA (MPA) or vigorous-intensity PA (VPA) and POP, except that women who had a history of hysterectomy were more likely to develop POP when performing more VPA (53% higher risk for every additional 15 min/day). Substituting 1 h/day of LPA with equivalent sedentary time was associated with a 18% (95% CI, 11%–24%) lower risk of POP. The risk can also be reduced by 17% (95% CI, 7%–25%) through substituting 30 min/day of LPA with MPA.

**Conclusions:**

More time spent in LPA or less sedentary time was linked to an elevated risk of POP in middle-aged and elderly women, while MPA or VPA was not. Substituting LPA with equivalent duration of sedentary behavior or MPA may lower the risk of POP.

**Supplementary Information:**

The online version contains supplementary material available at 10.1186/s12966-024-01559-w.

## Introduction

The pelvic floor, composed of muscles, fascia and ligaments, is a hammock-like structure that pockets the pelvic organs (bladder, uterus, rectum, etc.). Pelvic organ prolapse (POP) is the descent of pelvic organs towards or beyond the vagina opening when the pelvic floor becomes too weak to hold them in place. Symptoms of POP may include vaginal bulging, urinary/fecal incontinence, uncomfortable intercourse, etc. [1]. The prevalence of symptomatic POP increased from 1.8% in women aged twenties to 19.2% in women aged seventies or more [2]. The risk for POP surgery peaked at seventies with an annual risk of 4.3/1000 women and a lifetime risk of 12.6% [3]. POP not only seriously affects patients’ quality of life, but also causes a huge financial burden. About 1.52 billion dollars were spent per year on treating POP in the U.S. between 2016 and 2018 [4].

Advancing age, increasing parity, and previous hysterectomy are the most established risk factors for POP, yet difficult to be modified [5]. Physical activity (PA) is perceived as a potential modifiable factor to reduce the burden of POP, as it may influence the strength of the pelvic floor. However, related evidence is scarce and controversial. A cross-sectional study found that women engaged in high-impact PA (running, jumping, and ball sports) had a lower prevalence of symptomatic prolapse than those undertaking low-impact PA (walking, swimming, and light physical exercise), while another two observed no associations between PA and POP [6–8]. Since PA and POP were assessed at the same point in time in these studies, they could not determine whether PA influenced the risk of POP or the presence of POP altered the habit of PA. Though two case-control studies also reported no associations, they were limited by small sample sizes (< 400) and recall bias from self-reported PA. In prospective cohorts, a higher risk of POP was observed in soldiers after a 6-week paratrooper training and in women with a moderately high amount of PA at early postpartum after one year of follow-up. Whether this applies to the general population in a longer follow-up is unknown. In addition, dose-response analyses were absent and the impact of sedentary behavior, which accounts for most of our day time, has barely been investigated.

To fill the knowledge gaps, this study aimed to investigate the associations of objective accelerometer-measured PA and sedentary behavior with the risk of incident POP in the UK Biobank, one of the largest population-based prospective cohort to date.

## Methods

### Study design and participants

The UK Biobank is a prospective cohort approved by the North-West Multi-Center Research Ethics Committee (reference 11/NW/0382). More than 0.5 million participants aged 37–73 years were enrolled into this cohort between 2006 and 2010 when they were asked to complete a touch-screen questionnaire, have physical and functional measurements, and provide biological samples in 22 assessment centers throughout the UK [9]. Later, 0.24 million participants were randomly invited to have their PA and sedentary behavior measured by the accelerometers between May 2013 and December 2015 (baseline of this study), with a response rate of 44%. Devices were dispatched for 106,053 participants and, of these, data were available for 58,245 women. All participants provided electronic-signed consents. This study followed the Strengthening the Reporting of Observational Studies in Epidemiology (STROBE) reporting guideline (online Supplemental checklist).

### Accelerometer-measured physical activity and sedentary behavior

PA and sedentary behavior were monitored by the Axivity AX3 triaxial accelerometer on the dominant hand for 7 days when the sensor captured the acceleration at 100 Hz with a dynamic range of ± 8 gravities (1 gravity = 9.81 m per second squared) [10]. After excluding participants who had an unexpectedly large or small amount of PA (*N* = 2,674), who did not have sufficient wear time (< 3 days or not having data in each one-hour period of the 24-h cycle, *N* = 2,368), whose PA data were not well calibrated (*N* = 3), and who experienced the shift to daylight saving time (*N* = 2,356), 50,844 women were left with valid accelerometry data. More details about data collection and processing can be found elsewhere [11]. The duration of light-intensity PA (LPA), moderate-intensity PA (MPA), and vigorous-intensity PA (VPA) in minutes per week was determined as the time spent in 30 to 125 milligravities, > 125 to 400 milligravities, and > 400 milligravities intensity activity, respectively [12].

### Outcome ascertainment

Incident POP cases and diagnosis date were ascertained by data from three sources (hospital admission data 83.9%, primary care 12.0%, and self-report 4.1%), where POP was defined as N81 and N99.3 according to the International Classification of Diseases, 10th Revision (ICD-10), including urethrocele, cystocele, uterovaginal prolapse, vaginal enterocele, rectocele, prolapse of vaginal vault after hysterectomy, and other POP. The follow-up began on the date when the accelerometry was completed and ended on the diagnosis date of POP, the date of death extracted from the death certificates, or the end of follow-up (November 1, 2022), whichever came first. Participants with pre-existing POP before the start of the follow-up were excluded from the analyses (*N* = 2,991).

### Covariates ascertainment

Covariates were selected based on a pre-defined directed acyclic graph (online Supplemental Figure S1). Age was determined from birthdate (acquired from central registry, updated by participant) and the date when the accelerometry started (derived from the accelerometers). Information on the other covariates were obtained from the anthropometric measurements conducted by the trained nurses and the self-report questionnaire completed during the recruitment, including body mass index (BMI), body fat percentage, ethnicity, Townsend deprivation index (TDI), smoking status, menopause status, history of hysterectomy, and number of children. BMI was constructed as weight in kilograms divided by height in meters squared. The height was measured using the Seca 240 measuring rod, while the weight and the body fat percentage were assessed using the Tanita BC418MA body composition analyzer. TDI is a composite metric of material deprivation that incorporates unemployment, non-car ownership, non-home ownership, and household overcrowding, with higher values indicating lower socio-economic status [13]. TDI was assigned to each participant according to their postcode and the result of preceding national census. Details of these covariates can be found on the website of the UK Biobank.

### Statistical analyses

Baseline characteristics of the participants were described as means and standard deviations or medians and interquartile ranges for continuous variables, and numbers (percentages) for categorical variables.

First, dose-response associations of the duration of PA and sedentary behavior with the risk of incident POP were evaluated using restricted cubic splines. The reference values were set at the median and the knots at the 5th, 35th, 65th, and 95th percentiles of the duration. Second, the duration was categorized into five levels mainly referring to the current PA guideline (150–300 min/week of MPA and 75–150 min/week of VPA) to investigate the risk of POP for different levels of duration in a single intensity as well as that for various combinations of duration levels in different intensities using Cox proportional hazards models. The proportional hazard assumption was checked with Schoenfeld residuals and no violation was observed. Linear trends were examined by entering the median value of each category of duration as a continuous variable into the models. Third, isotemporal substitution models were used to estimate the effects of substituting a type of activity with equivalent duration of others on the risk of POP. All analyses were adjusted for the following potential confounders: age (continuous), BMI (continuous), body fat percentage (continuous), ethnicity (white, Black or Black British, Asian or Asian British, or mixed), TDI (continuous), smoking status (never, previous, or current), menopause status (yes, no, not sure-had a hysterectomy, or not sure-other reason), history of hysterectomy (yes or no), and number of children (0, 1, or ≥ 2). Missing values in these covariates (< 1%) were imputed with mode values for categorical variables and median values for continuous variables.

Stratified analyses were conducted according to age (< 60 or ≥ 60 years), BMI (< 25 kg/m^2^ or ≥ 25 kg/m^2^), menopause status (yes or no), history of hysterectomy (yes or no), and number of children (0 or ≥ 1) to examine whether the associations varied by these factors. Interactions were tested by a likelihood-ratio test comparing models with and without product terms between PA or sedentary behavior and these factors.

Several sensitivity analyses were carried out to assess the robustness of our results. First, we excluded participants who were diagnosed with POP within the first 2 years of follow-up to avoid the potential risk of reverse causation. Second, competing risk models were applied to account for the competing risk of death. Third, LPA, MPA and VPA durations were mutually adjusted to evaluate whether the associations were attributable to other intensities. Fourth, we only included participants without missing covariates.

All the analyses were conducted using the R software (version 4.1.2). The statistical significance was set as *P* < 0.05 (two-sided test).

## Results

**Fig. 1:**
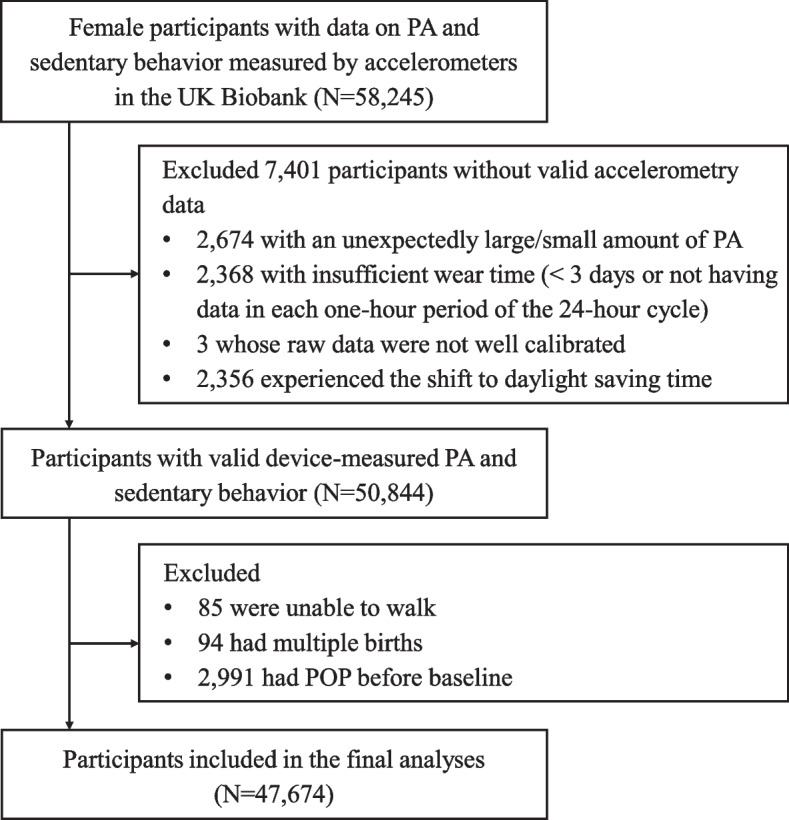
Flowchart. Abbreviation: PA, physical activity; POP, pelvic organ prolapse

Of the 502,488 UK Biobank participants, 58,245 women had their PA and sedentary behavior monitored by accelerometers. After excluding 7,401 participants without valid accelerometry data, 2,991 who had pre-existing POP, 85 who were unable to walk, and 94 who once had multiple births, 47,674 participants were left in the final analyses (Fig. 1). The median follow-up period was 8.0 years. The overall incidence rate of POP was 20.9 per 10,000 person-years (779 cases/372,079 person-years).

Participants included in the analyses were less likely to be obese, to smoke, or to have a history of hysterectomy at recruitment compared with the excluded (online Supplemental Table S1). Participants who developed POP during the follow-up were older, were more likely to be obese, to be in menopause, and to have a history of hysterectomy, and had more children at recruitment than those who did not (Table 1).

**Table 1:** Characteristics of 47,674 participants by presence of POP

Characteristics	Presence of POP		
	**No**	**Yes**	**Overall**
**Number**	46,895	779	47,674
**Age at accelerometry (years)**			
Median (IQR)	61.9 (12.3)	64.8 (9.4)	61.9 (12.2)
**Ethnicity, n (%)**			
White	45,187 (96.4)	766 (98.3)	45,953 (96.4)
Mixed	610 (1.3)	8 (1.0)	618 (1.3)
Asian or Asian British	511 (1.1)	3 (0.4)	514 (1.1)
Black or Black British	449 (1.0)	2 (0.3)	451 (0.9)
Missing	138 (0.3)	0 (0)	138 (0.3)
**TDI**			
Median (IQR)	-2.4 (3.7)	-2.6 (3.5)	-2.4 (3.7)
**BMI, kg/m**^**2**^**, n (%)**			
< 25	22,048 (47.0)	286 (36.7)	22,334 (46.8)
25 ~ 29.9	16,126 (34.4)	320 (41.1)	16,446 (34.5)
≥ 30	8,336 (17.8)	170 (21.8)	8,506 (17.8)
Missing	385 (0.8)	3 (0.4)	388 (0.8)
**Body fat percentage (%)**			
Mean (SD)	35.3 (6.8)	36.9 (6.2)	35.3 (6.8)
**Smoking, n (%)**			
Never	28,592 (61.0)	474 (60.8)	29,066 (61.0)
Previous	15,381 (32.8)	267 (34.3)	15,648 (32.8)
Current	2,799 (6.0)	37 (4.7)	2,836 (5.9)
Missing	123 (0.3)	1 (0.1)	124 (0.3)
**Number of children, n (%)**			
0	10,768 (23.0)	37 (4.7)	10,805 (22.7)
1	6,102 (13.0)	85 (10.9)	6,187 (13.0)
≥ 2	29,987 (63.9)	657 (84.3)	30,644 (64.3)
Missing	38 (0.1)	0 (0)	38 (0.1)
**Menopause, n (%)**			
No	12,502 (26.7)	129 (16.6)	12,631 (26.5)
Yes	27,512 (58.7)	515 (66.1)	28,027 (58.8)
Not sure	6,822 (14.5)	135 (17.3)	6,957 (14.6)
Missing	59 (0.1)	0 (0)	59 (0.1)
**History of hysterectomy, n (%)**			
No	40,006 (85.3)	619 (79.5)	40,625 (85.2)
Yes	6,795 (14.5)	154 (19.8)	6,949 (14.6)
Missing	94 (0.2)	6 (0.8)	100 (0.2)

Continuous variables were presented as mean (standard deviation) if normally distributed and as median (interquartile range) otherwise

*Abbreviations*: *BMI* Body mass index, *IQR* Interquartile range, *POP* Pelvic organ prolapse, *SD* Standard deviation, *TDI* Townsend deprivation index

**Fig. 2:**
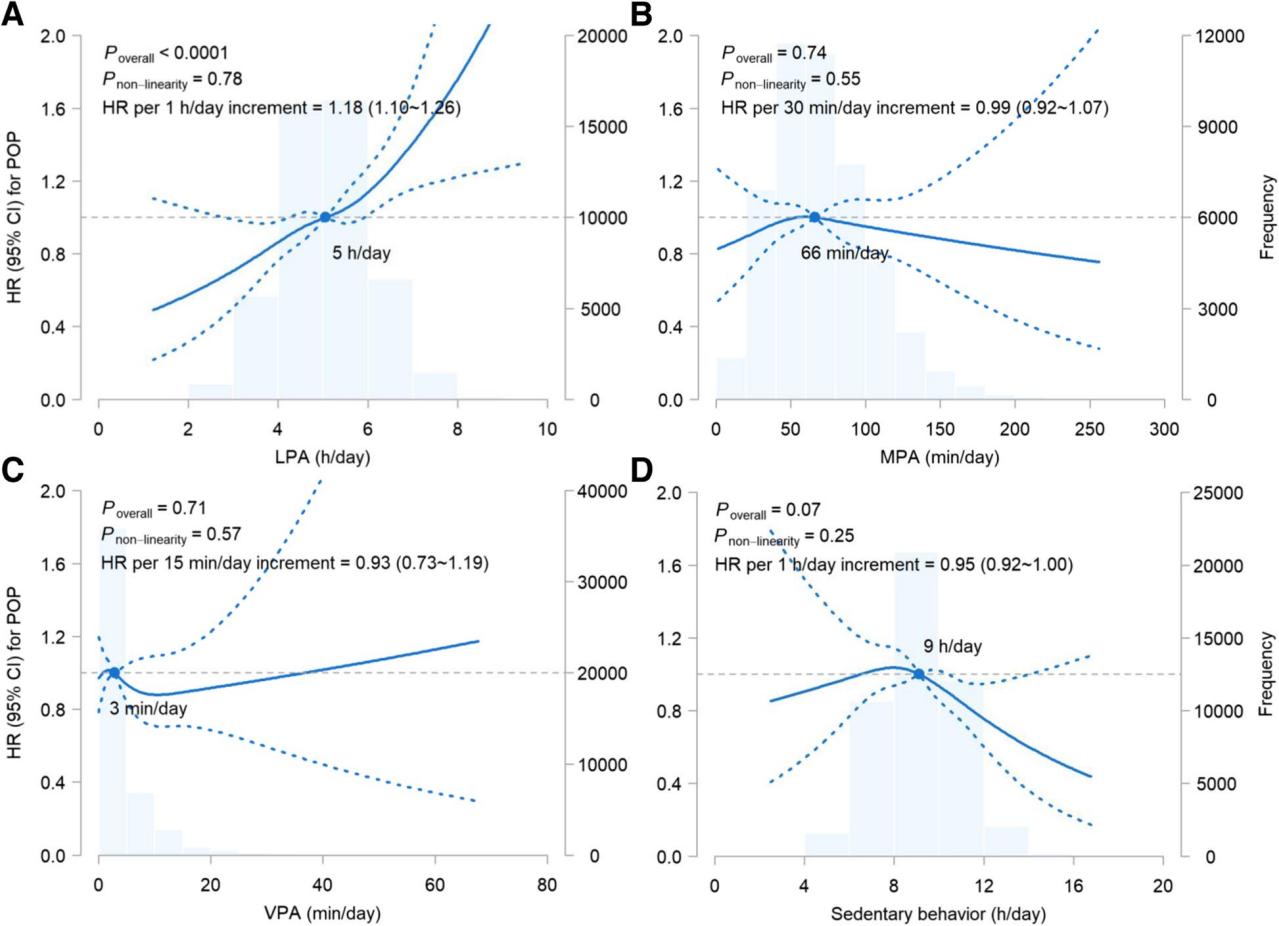
Dose-response associations of PA and sedentary behavior with the risk of POP. Abbreviations: CI, confidence interval; h, hour; HR, hazard ratio; LPA, light-intensity physical activity; min, minute; MPA, moderate-intensity physical activity; PA, physical activity; POP, pelvic organ prolapse; VPA, vigorous-intensity physical activity

There was a positive linear association between LPA and POP after multivariable adjustment (Fig. 2). Every additional 1 h/day of LPA elevated the risk of POP by 18% (95% confidence interval [CI], 10%–26%; *P* < 0.001). Compared with < 4 h/day of LPA, the hazard ratios (HRs) of POP increased as more time was spent in LPA (1.23 [95% CI, 0.96–1.58] for 4 ~  < 5 h/day, 1.31 [1.03–1.68] for 5 ~  < 6 h/day, 1.62 [1.23–2.13] for 6 ~  < 7 h/day, and 1.86 [1.26–2.75] for ≥ 7 h/day; *P*_trend_ < 0.001; Table 2). No associations were observed between the duration of MPA or VPA and the risk of POP. More time spent in sedentary behavior, especially beyond the average level (9 h/day), was linked to a lower risk of POP. The HR per 1 h/day increment was 0.95 (95% CI, 0.92–1.00).

**Table 2:** Association of PA and sedentary behavior with risks of POP

Duration of Activity		Cases/Person-years	Crude HR (95% CI)	Adjusted HR (95% CI)	*P* for trend
LPA (h/day)	< 4	86/51,172	1 (Reference)	1 (Reference)	< 0.001
	4 ~ < 5	255/127,160	1.20 (0.94 to 1.53)	1.23 (0.96 to 1.58)	
	5 ~ < 6	264/126,652	1.25 (0.98 to 1.59)	1.31 (1.03 to 1.68)	
	6 ~ < 7	137/54,007	1.52 (1.16 to 1.99)	1.62 (1.23 to 2.13)	
	≥ 7	37/13,088	1.69 (1.15 to 2.48)	1.86 (1.26 to 2.75)	
MPA (min/day)	< 30	75/30,608	1 (Reference)	1 (Reference)	0.91
	30 ~ < 60	297/124,630	0.99 (0.76 to 1.27)	1.16 (0.90 to 1.50)	
	60 ~ < 90	251/123,393	0.84 (0.65 to 1.09)	1.15 (0.88 to 1.50)	
	90 ~ < 120	109/62,508	0.73 (0.54 to 0.97)	1.10 (0.80 to 1.50)	
	≥ 120	47/30,939	0.63 (0.44 to 0.91)	1.05 (0.72 to 1.55)	
VPA (min/day)	0	158/62,639	1 (Reference)	1 (Reference)	0.77
	> 0 ~ < 5	469/216,292	0.87 (0.72 to 1.04)	1.03 (0.86 to 1.24)	
	5 ~ < 15	125/76,725	0.66 (0.52 to 0.83)	0.96 (0.75 to 1.23)	
	15 ~ < 30	22/13,508	0.66 (0.42 to 1.02)	1.10 (0.70 to 1.75)	
	≥ 30	5/2,916	0.69 (0.28 to 1.69)	1.35 (0.55 to 3.33)	
Sedentary behavior (h/day)	< 7	82/41,034	1 (Reference)	1 (Reference)	0.15
	7 ~ < 8	125/55,033	1.13 (0.86 to 1.49)	1.06 (0.80 to 1.40)	
	8 ~ < 9	174/79,676	1.09 (0.84 to 1.42)	1.00 (0.77 to 1.31)	
	9 ~ < 10	176/83,861	1.05 (0.80 to 1.36)	0.96 (0.73 to 1.24)	
	≥ 10	222/112,475	0.98 (0.76 to 1.26)	0.89 (0.69 to 1.15)	

The model was adjusted for age when the accelerometry started (year, continuous), ethnicity (White, Black or Black British, Asian or Asian British, or mixed), Townsend deprivation index (continuous), body mass index (kg/m^2^, continuous), body fat percentage (%, continuous), smoking status (never, previous, or current), menopause status (yes, no, not sure with a history of hysterectomy, or not sure with other reasons), history of hysterectomy (yes or no), and number of births (0, 1, or ≥ 2)

*Abbreviations*: *CI* Confidence interval, *h* Hour, *HR* Hazard ratio, *LPA* Light-intensity physical activity, *min* Minute, *MPA* Moderate-intensity physical activity, *PA* Physical activity, *POP* Pelvic organ prolapse, *VPA* Vigorous-intensity physical activity

**Fig. 3:**
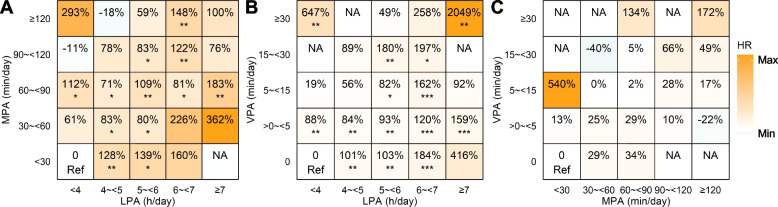
Joint associations of PA and the risk of POP. Abbreviations: h, hour; LPA, light-intensity physical activity; min, minute; MPA, moderate-intensity physical activity; NA, not available; PA, physical activity; POP, pelvic organ prolapse; Ref, reference; VPA, vigorous-intensity physical activity. * < 0.05, ** < 0.01, *** < 0.001. The percentages indicate the increased risk of POP in each combination of PA compared with the least active group

The additional risk of POP in participants undertaking various combinations of PA compared to the least active ones is shown in Fig. 3. The HR data used to generate this figure are presented in online Supplemental Tables S2-S4. Though more LPA was associated with an elevated risk of POP, adding more MPA or VPA to a high level of LPA did not necessarily further increase the risk. For example, compared with the combination of < 4 h/day of LPA and < 30 min/day of MPA, 5 ~  < 6 h/day of LPA was associated with 139%, 80%, 109%, 83%, and 59% higher risks of POP when combined with < 30, 30 ~  < 60, 60 ~  < 90, 90 ~  < 120, and ≥ 120 min/day of MPA.

Substituting 1 h/day of LPA with equivalent sedentary time was associated with a 18% (95% CI, 11%–24%) lower risk of POP. The risk can also be reduced by 17% (95% CI, 7%–25%) through substituting 30 min/day of LPA with equivalent duration of MPA (Table 3). In contrast, substitution of LPA with VPA was not associated with POP (HR, 0.97; 95% CI, 0.74–1.27).

**Table 3:** HR for POP when substituting LPA with equivalent duration of other types of activity

Substitution	Crude HR (95% CI)	Adjusted HR (95% CI)
LPA → Sedentary behavior (1 h/day)	0.80 (0.74 to 0.86)	0.82 (0.76 to 0.89)
LPA → MPA (30 min/day)	0.74 (0.66 to 0.82)	0.83 (0.75 to 0.93)
LPA → VPA (15 min/day)	0.78 (0.59 to 1.02)	0.97 (0.74 to 1.27)

The model was adjusted for age when the accelerometry started (year, continuous), ethnicity (White, Black or Black British, Asian or Asian British, or mixed), Townsend deprivation index (continuous), body mass index (kg/m^2^, continuous), body fat percentage (%, continuous), smoking status (never, previous, or current), menopause status (yes, no, not sure with a history of hysterectomy, or not sure with other reasons), history of hysterectomy (yes or no), and number of births (0, 1, or ≥ 2)

*Abbreviations*: *CI* Confidence interval, *h* Hour, *HR* Hazard ratio, *LPA* Light-intensity physical activity, *min* Minute, *MPA* Moderate-intensity physical activity, *POP* Pelvic organ prolapse, *VPA* Vigorous-intensity physical activity

No interactions were observed between any intensity of PA or sedentary behavior and age, BMI, menopause status, or number of children (online Supplemental Figures S2-S5). Every additional 15 min/day of VPA elevated the risk of POP by 53% (95% CI, -0.1%–136%) in women with a history of hysterectomy, but no association was found between VPA and POP in women without this history (*P*_interaction_ = 0.02). Results of four sensitivity analyses were similar to those of the main analyses (online Supplemental Tables S5-S8 and Figures S6-S8).

## Discussion

In this large population-based cohort study, either more time spent in LPA or less time spent in sedentary behavior was observed to elevate the risk of POP among middle-aged and elderly women. Duration of MPA or VPA was not associated with the risk of POP. Substituting LPA with equivalent duration of sedentary behavior or MPA might reduce the risk.

### Comparison with other studies

LPA refers to activities that require low levels of energy expenditure (1.5–3 metabolic equivalent of task, METs), such as walking very slowly, doing household chores, or standing [14]. No studies have directly investigated the relationship between LPA and POP. A case-control study found that housewives were at a three times higher risk of urogenital prolapse than professional/managerial women [15]. Though this study did not collect information about PA, another study focusing on examining PA patterns across different occupations reported that participants keeping house had the most LPA (6.3 h/day) among all occupations studied and most of their time in LPA were spent in food preparation and serving [16]. This result is consistent with ours to some extent. In our study, the risk of POP increased as more time was spent in LPA. Participants who engaged in ≥ 7 h/day of LPA were at a 1.86 times higher risk of POP than those who engaged in < 4 h/day of LPA. The main between-study difference is that our results were based on accelerometer-measured PA in a prospective cohort study and were not limited to people who were involved in certain jobs.

MPA refers to PA that is performed between 3 and < 6 times the intensity of rest (METs), such as walking at a brisk pace, general home exercises, and dancing; while PA that is carried out at 6 or more METs is defined as VPA, such as jogging, most competitive sports, and heavy housework [17, 18]. Results of previous studies on the association of MPA or VPA with POP were mixed. An early adulthood history of competitive sports, current PA, or lifetime VPA were not observed to associate with the risk of POP [19–21]. In contrast, several studies reported associations between heavy occupational PA, particularly heavy lifting, and POP [22–24]. These studies were in cross-sectional or case-control design with limited sample sizes based on questionnaire-derived self-reported PA, while the lack of association observed in our study was based on accelerometer-measured PA in a prospective population-based cohort.

Our finding that substituting LPA with equivalent sedentary time reduced the risk of POP was different from that in another prospective cohort study, which found that decreasing sedentary time by 30 min/day with a concomitant increase in LPA at six months postpartum was not associated with the prevalence of worse pelvic floor support at one year postpartum in primiparas delivered vaginally [25]. This study may not be comparable to ours because the level of PA and the status of pelvic floor at postpartum in women aged around 30 years are likely to be different from those in women aged around 60 years.

### Result interpretation and research implications

The pelvic floor forms a hammock-like structure that pockets the pelvic organs. The downward forces the pelvic floor needs to withstand include the gravity of the pelvic organs and the intra-abdominal pressure, which can be increased by PA. LPAs are generally considered safe and beneficial for health, as they can help reduce the risk of chronic diseases and promote mental well-being [26–28]. However, engaging in LPA for a long period of time, such as standing for several hours or doing repetitive tasks, may cause a stretch to the muscles and the ligaments of the pelvic floor, leading to muscle fatigue and chronic tissue damage, which increases the risk of POP. Results of the upright computed tomography indicated that the pelvic floor descended significantly in the standing position, compared with the supine position [29]. The total duration of LPA is usually quite long. In this study, the median duration of LPA was 5 h/day, same as that in South Asian, but longer than that (4 h/day) in Americans [14, 30]. The impact of LPA on the pelvic floor is gentle but cumulative. People may not be sufficiently aware of the adverse impacts of LPA because severe symptoms are unlikely to be observed in a short term and the long duration of all LPAs can be easily ignored because most LPAs are fragmented daily activities. The risk of POP might be reduced by substituting LPA with sedentary behavior or MPA. Wearable devices equipped with accelerometers may help monitor and modify the duration and intensity of PA. Related evidence and recommendations are warranted in future guidelines.

Similar to the case of LPA, a long duration might also be a requisite for MPA and VPA to result in the damage of the pelvic floor. A prior study found that though the maximum vertical ground reaction forces were the largest for long jumping (16 times bodyweight), followed by javelin throwing (9 times), and running (4 times), the majority of female athletes suffering from urinary incontinence were long-distance runners (32.1%), followed by middle-distance runners (25.0%) and those who participated in speed events (17.9%), throwing events (14.3%), and jumping events (10.7%) [31, 32]. This indicates that the duration of force on the pelvic floor may play a more important role than the magnitude of force in causing pelvic floor damage. For the general population, MPA and VPA are mostly exercises that do not last long, especially as people age. The median duration of MPA and VPA was 66 min/day and 3 min/day in our study, while the average duration of moderate-to-vigorous-intensity PA in participants with similar age was comparable in China (69 min/day), but much shorter in the U.S. (21 min/day) and Canada (18 min/day) [14, 33, 34]. MPA or VPA of this duration may not reach the threshold for increasing the risk of POP. Therefore, middle-aged and elderly women without contraindications are encouraged to perform MPA and VPA as the PA guideline suggests to obtain other potential health benefits [17]. One exception is that women who had a history of hysterectomy were more likely to develop POP when performing a longer duration of VPA, probably because hysterectomy may damage or weaken the pelvic floor muscles and ligaments, making them more vulnerable to VPA.

### Strengths and limitations

To our knowledge, this is the first study that comprehensively examined the long-term association of PA and sedentary behavior with the risk of POP in a large-scale population-based prospective cohort. Accelerometry data is robust against recall bias and can reduce the probability of leaving out fragmented PA, especially LPA. More than 95% of POP cases were ascertained by hospital or primary care records following the ICD-10. Information on a wide range of covariates facilitated confounding adjustment. However, this study has some limitations that need to be considered. Firstly, we were unable to differentiate PA from different domains (i.e., recreational PA like sports and exercise, non-recreational PA like PA occurring during transportation or work, housework, and yardwork). This information can only be obtained from self-report questionnaires and is therefore subject to recall bias. Besides, the duration and intensity of PA may vary a lot in the same occupation, it is the actual amount of PA, instead of some certain types of job, that finally determines the risk of POP. In addition, information on all covariates except age were collected at recruitment instead of at accelerometer wear date. There may be variation in the status of some covariates (BMI, body fat percentage, menopause status, history of hysterectomy) in some participants during this period. Another limitation is that PA was only measured once. A longitudinal study reported that PA was reasonably stable over six time points spanning ten years in older adults in England, though there was an overall trend for increasing levels of inactivity and a reduction in VPA [35]. If this measurement does not represent habitual PA levels, underestimation of risk associations could occur [36]. As for any observational studies, we cannot rule out the possibility of residual/unmeasured confounding and reverse causation. Lastly, the UK Biobank had a response rate of 5.5% and it was not representative of the general UK population, but a previous study showed that risk factor associations in this cohort were comparable to those from representative studies [37].

## Conclusions

A long duration may be a requisite for PA to increase the risk of POP. In middle-aged and elderly women, more time spent in LPA or less time spent in sedentary behavior was associated with an elevated risk of POP, while MPA or VPA was not. To reduce the risk of POP, women who undertake too much LPA are recommended to substitute LPA with sedentary behavior or MPA. This is of great public health importance particularly given the increasing number of POP as the population ages.

### Supplementary Information

**Additional file 1: Checklist**. Strengthening the Reporting of Observational Studies in Epidemiology (STROBE). **Figure S1**. Directed acyclic graph to guide covariate selection. **Figure S2**. Stratified analyses of the association between LPA (per 1 h/day increment) and the risk of POP. **Figure S3**. Stratified analyses of the association between MPA (per 30 min/day increment) and the risk of POP. **Figure S4**. Stratified analyses of the association between VPA (per 15 min/day increment) and the risk of POP. **Figure S5**. Stratified analyses of the association between sedentary behavior (per 1h/day increment) and the risk of POP. **Figure S6**. Dose-response association of PA and sedentary behavior with the risk of POP after excluding participants who were diagnosed with POP in the first two years of follow-up (*N*=47,190). **Figure S7**. Dose-response association between PA and the risk of POP with mutual adjustment (*N*=47,674). **Figure S8**. Dose-response association of PA and sedentary behavior with the risk of POP in complete cases (*N*=46,283). **Table S1**. Participant characteristics by inclusion status. **Table S2**. Joint association of LPA and MPA with the risk of POP. **Table S3**. Joint association of LPA and VPA with the risk of POP. **Table S4**. Joint association of MPA and VPA with the risk of POP. **Table S5**. Association of PA and sedentary behavior with the risk of POP after excluding participants who were diagnosed with POP in the first two years of follow-up (*N*=47,190). **Table S6**. Association of PA and sedentary behavior with the risk of POP in competing risk models (*N*=47,674). **Table S7**. Association between PA and the risk of POP with mutual adjustment (*N*=47,674). **Table S8**. Association of PA and sedentary behavior with the risk of POP in complete cases (*N*=46,283).

## Data Availability

Data may be obtained from a third party and are not publicly available. Data are owned by the UK Biobank study. Qualified researchers can access the data by completing a short application form and paying a data access fee.

## Abbreviations

BMI: Body mass index
CI: Confidence interval
h: Hour
HR: Hazard ratio
ICD-10: International Classification of Diseases, 10th Revision
LPA: Light-intensity physical activity
MET: Metabolic equivalent of task
min: Minute
MPA: Moderate-intensity physical activity
PA: Physical activity
POP: Pelvic organ prolapse
STROBE: Strengthening the Reporting of Observational Studies in Epidemiology
TDI: Townsend deprivation index
UK: United Kingdom
U.S.: United States
VPA: Vigorous-intensity physical activity

## References

[1] Barber MD (2016). Pelvic organ prolapse. BMJ (Clinical research ed).

[2] Pang H, Zhang L, Han S, Li Z, Gong J, Liu Q, Liu X, Wang J, Xia Z, Lang J (2021). A nationwide population-based survey on the prevalence and risk factors of symptomatic pelvic organ prolapse in adult women in China - a pelvic organ prolapse quantification system-based study. BJOG.

[3] Wu JM, Matthews CA, Conover MM, Pate V, Jonsson Funk M (2014). Lifetime risk of stress urinary incontinence or pelvic organ prolapse surgery. Obstet Gynecol.

[4] St Martin B, Markowitz MA, Myers ER, Lundsberg LS, Ringel N. Estimated national cost of pelvic organ prolapse surgery in the United States. Obstet Gynecol. 2023. Online ahead of print. 10.1097/AOG.0000000000005485 38128098

[5] Iglesia CB, Smithling KR (2017). Pelvic organ prolapse. Am Fam Physician.

[6] Nygaard IE, Wolpern A, Bardsley T, Egger MJ, Shaw JM (2021). Early postpartum physical activity and pelvic floor support and symptoms 1 year postpartum. Am J Obstet Gynecol.

[7] Larsen WI, Yavorek T (2007). Pelvic prolapse and urinary incontinence in nulliparous college women in relation to paratrooper training. Int Urogynecol J Pelvic Floor Dysfunct.

[8] Miedel A, Tegerstedt G, Mæhle-Schmidt M, Nyrén O, Hammarström M (2009). Nonobstetric risk factors for symptomatic pelvic organ prolapse. Obstet Gynecol.

[9] Sudlow C, Gallacher J, Allen N, Beral V, Burton P, Danesh J, Downey P, Elliott P, Green J, Landray M (2015). UK biobank: an open access resource for identifying the causes of a wide range of complex diseases of middle and old age. PLoS Med.

[10] Khurshid S, Weng LC, Nauffal V, Pirruccello JP, Venn RA, Al-Alusi MA, Benjamin EJ, Ellinor PT, Lubitz SA (2022). Wearable accelerometer-derived physical activity and incident disease. NPJ Digital Medicine.

[11] Strain T, Wijndaele K, Dempsey PC, Sharp SJ, Pearce M, Jeon J, Lindsay T, Wareham N, Brage S (2020). Wearable-device-measured physical activity and future health risk. Nat Med.

[12] Ho FK, Zhou Z, Petermann-Rocha F, Para-Soto S, Boonpor J, Welsh P, Gill JMR, Gray SR, Sattar N, Pell JP (2022). Association between device-measured physical activity and incident heart failure: a prospective cohort study of 94 739 UK Biobank participants. Circulation.

[13] Nagar SD, Nápoles AM, Jordan IK, Mariño-Ramírez L (2021). Socioeconomic deprivation and genetic ancestry interact to modify type 2 diabetes ethnic disparities in the United Kingdom. EClinicalMedicine.

[14] Del Pozo CB, Biddle SJH, Gardiner PA, Ding D (2021). Light-intensity physical activity and life expectancy: national health and nutrition survey. Am J Prev Med.

[15] Chiaffarino F, Chatenoud L, Dindelli M, Meschia M, Buonaguidi A, Amicarelli F, Surace M, Bertola E, Di Cintio E, Parazzini F (1999). Reproductive factors, family history, occupation and risk of urogenital prolapse. Eur J Obstet Gynecol Reprod Biol.

[16] Quinn TD, Pettee Gabriel K, Siddique J, Aaby D, Whitaker KM, Lane-Cordova A, Sidney S, Sternfield B, Barone Gibbs B (2020). Sedentary time and physical activity across occupational classifications. Am J Health Promot.

[17] Bull FC, Al-Ansari SS, Biddle S, Borodulin K, Buman MP, Cardon G, Carty C, Chaput JP, Chastin S, Chou R (2020). World Health Organization 2020 guidelines on physical activity and sedentary behaviour. Br J Sports Med.

[18] General Physical Activities Defined by Level of Intensity. https://www.cdc.gov/nccdphp/dnpa/physical/pdf/PA_Intensity_table_2_1.pdf.

[19] Nygaard IE, Shaw JM, Bardsley T, Egger MJ (2014). Lifetime physical activity and pelvic organ prolapse in middle-aged women. Am J Obstet Gynecol.

[20] Kuutti MA, Hyvärinen M, Kauppinen M, Sipilä S, Aukee P, Laakkonen EK (2023). Early adulthood and current physical activity and their association with symptoms of pelvic floor disorders in middle-aged women: An observational study with retrospective physical activity assessment. BJOG.

[21] Hendrix SL, Clark A, Nygaard I, Aragaki A, Barnabei V, McTiernan A (2002). Pelvic organ prolapse in the Women's Health Initiative: gravity and gravidity. Am J Obstet Gynecol.

[22] Braekken IH, Majida M, EllströmEngh M, Holme IM, Bø K (2009). Pelvic floor function is independently associated with pelvic organ prolapse. BJOG.

[23] Jørgensen S, Hein HO, Gyntelberg F (1994). Heavy lifting at work and risk of genital prolapse and herniated lumbar disc in assistant nurses. Occup Med (Oxford, England).

[24] Woodman PJ, Swift SE, O'Boyle AL, Valley MT, Bland DR, Kahn MA, Schaffer JI (2006). Prevalence of severe pelvic organ prolapse in relation to job description and socioeconomic status: a multicenter cross-sectional study. Int Urogynecol J Pelvic Floor Dysfunct.

[25] Shaw JM, Wolpern A, Wu J, Nygaard IE, Egger MJ (2023). Postpartum sedentary behaviour and pelvic floor support: A prospective cohort study. J Sports Sci.

[26] Füzéki E, Engeroff T, Banzer W (2017). Health benefits of light-intensity physical activity: a systematic review of accelerometer data of the National Health and Nutrition Examination Survey (NHANES). Sports Med (Auckland, NZ).

[27] Chastin SFM, De Craemer M, De Cocker K, Powell L, Van Cauwenberg J, Dall P, Hamer M, Stamatakis E (2019). How does light-intensity physical activity associate with adult cardiometabolic health and mortality? Systematic review with meta-analysis of experimental and observational studies. Br J Sports Med.

[28] Amagasa S, Machida M, Fukushima N, Kikuchi H, Takamiya T, Odagiri Y, Inoue S (2018). Is objectively measured light-intensity physical activity associated with health outcomes after adjustment for moderate-to-vigorous physical activity in adults? A systematic review. Int J Behav Nutr Phys Act.

[29] Jinzaki M, Yamada Y, Nagura T, Nakahara T, Yokoyama Y, Narita K, Ogihara N, Yamada M (2020). Development of upright computed tomography with area detector for whole-body scans: phantom study, efficacy on workflow, effect of gravity on human body, and potential clinical impact. Invest Radiol.

[30] Biddle GJH, Edwardson CL, Rowlands AV, Davies MJ, Bodicoat DH, Hardeman W, Eborall H, Sutton S, Griffin S, Khunti K (2019). Differences in objectively measured physical activity and sedentary behaviour between white Europeans and south Asians recruited from primary care: cross-sectional analysis of the PROPELS trial. BMC Public Health.

[31] Hay JG (1993). Citius, altius, longius (faster, higher, longer): the biomechanics of jumping for distance. J Biomech.

[32] Velázquez-Saornil J, Méndez-Sánchez E, Gómez-Sánchez S, Sánchez-Milá Z, Cortés-Llorente E, Martín-Jiménez A, Sánchez-Jiménez E, Campón-Chekroun A (2021). Observational Study on the Prevalence of Urinary Incontinence in Female Athletes. Int J Environ Res Public Health.

[33] Cerin E, Van Dyck D, Zhang CJP, Van Cauwenberg J, Lai PC, Barnett A (2020). Urban environments and objectively-assessed physical activity and sedentary time in older Belgian and Chinese community dwellers: potential pathways of influence and the moderating role of physical function. Int J Behav Nutr Phys Act.

[34] Clarke J, Colley R, Janssen I, Tremblay MS (2019). Accelerometer-measured moderate-to-vigorous physical activity of Canadian adults, 2007 to 2017. Health Rep.

[35] Smith L, Gardner B, Fisher A, Hamer M (2015). Patterns and correlates of physical activity behaviour over 10 years in older adults: prospective analyses from the English Longitudinal Study of Ageing. BMJ Open.

[36] Clarke R, Shipley M, Lewington S, Youngman L, Collins R, Marmot M, Peto R (1999). Underestimation of risk associations due to regression dilution in long-term follow-up of prospective studies. Am J Epidemiol.

[37] Batty GD, Gale CR, Kivimaki M, Deary IJ, Bell S (2020). Comparison of risk factor associations in UK Biobank against representative, general population based studies with conventional response rates: prospective cohort study and individual participant meta-analysis. BMJ.

