# Supplementary material for: Accelerometer-measured physical activity, sedentary behavior, and risk of incident pelvic organ prolapse: a prospective cohort study in the UK Biobank
Source: Int J Behav Nutr Phys Act. 2024 Feb 2;21:12. doi: 10.1186/s12966-024-01559-w (PMC10835866; doi:10.1186/s12966-024-01559-w)
Supplement: Supplementary file 1 — Additional file 1: Checklist. Strengthening the Reporting of Observational Studies in Epidemiology (STROBE). Figure S1. Directed acyclic graph to guide covariate selection. Figure S2. Stratified analyses of the association between LPA (per 1 h/day increment) and the risk of POP. Figure S3. Stratified analyses of the association between MPA (per 30 min/day increment) and the risk of POP. Figure S4. Stratified analyses of the association between VPA (per 15 min/day increment) and the risk of POP. Figure S5. Stratified analyses of the association between sedentary behavior (per 1h/day increment) and the risk of POP. Figure S6. Dose-response association of PA and sedentary behavior with the risk of POP after excluding participants who were diagnosed with POP in the first two years of follow-up (N=47,190). Figure S7. Dose-response association between PA and the risk of POP with mutual adjustment (N=47,674). Figure S8. Dose-response association of PA and sedentary behavior with the risk of POP in complete cases (N=46,283). Table S1. Participant characteristics by inclusion status. Table S2. Joint association of LPA and MPA with the risk of POP. Table S3. Joint association of LPA and VPA with the risk of POP. Table S4. Joint association of MPA and VPA with the risk of POP. Table S5. Association of PA and sedentary behavior with the risk of POP after excluding participants who were diagnosed with POP in the first two years of follow-up (N=47,190). Table S6. Association of PA and sedentary behavior with the risk of POP in competing risk models (N=47,674). Table S7. Association between PA and the risk of POP with mutual adjustment (N=47,674). Table S8. Association of PA and sedentary behavior with the risk of POP in complete cases (N=46,283). [file 12966_2024_1559_MOESM1_ESM.docx]

**Supplemental Materials**

**Checklist**. Strengthening the Reporting of Observational Studies in Epidemiology (STROBE)

**Figure S1**. Directed acyclic graph to guide covariate selection

**Figure S2**. Stratified analyses of the association between LPA (per 1 h/day increment) and the risk of POP

**Figure S3**. Stratified analyses of the association between MPA (per 30 min/day increment) and the risk of POP

**Figure S4**. Stratified analyses of the association between VPA (per 15 min/day increment) and the risk of POP

**Figure S5**. Stratified analyses of the association between sedentary behavior (per 1h/day increment) and the risk of POP

**Figure S6**. Dose-response association of PA and sedentary behavior with the risk of POP after excluding participants who were diagnosed with POP in the first two years of follow-up (N=47,190)

**Figure S7**. Dose-response association between PA and the risk of POP with mutual adjustment (N=47,674)

**Figure S8**. Dose-response association of PA and sedentary behavior with the risk of POP in complete cases (N=46,283)

**Table S1**. Participant characteristics by inclusion status

**Table S2**. Joint association of LPA and MPA with the risk of POP

**Table S3**. Joint association of LPA and VPA with the risk of POP

**Table S4**. Joint association of MPA and VPA with the risk of POP

**Table S5**. Association of PA and sedentary behavior with the risk of POP after excluding participants who were diagnosed with POP in the first two years of follow-up (N=47,190)

**Table S6**. Association of PA and sedentary behavior with the risk of POP in competing risk models (N=47,674)

**Table S7**. Association between PA and the risk of POP with mutual adjustment (N=47,674)

**Table S8**. Association of PA and sedentary behavior with the risk of POP in complete cases (N=46,283)

**Checklist**. Strengthening the Reporting of Observational Studies in Epidemiology (STROBE)

|  | Item No | Recommendation | Page No |
| --- | --- | --- | --- |
| **Title and abstract** | 1 | (*a*) Indicate the study’s design with a commonly used term in the title or the abstract | 1, 2 |
|  |  | (*b*) Provide in the abstract an informative and balanced summary of what was done and what was found | 2 |
| Introduction | | | |
| Background/rationale | 2 | Explain the scientific background and rationale for the investigation being reported | 3 |
| Objectives | 3 | State specific objectives, including any prespecified hypotheses | 3, 4 |
| Methods | | | |
| Study design | 4 | Present key elements of study design early in the paper | 4 |
| Setting | 5 | Describe the setting, locations, and relevant dates, including periods of recruitment, exposure, follow-up, and data collection | 4 |
| Participants | 6 | (*a*) Give the eligibility criteria, and the sources and methods of selection of participants. Describe methods of follow-up | 4, Fig 1 |
|  |  | (*b*) For matched studies, give matching criteria and number of exposed and unexposed | Not applicable |
| Variables | 7 | Clearly define all outcomes, exposures, predictors, potential confounders, and effect modifiers. Give diagnostic criteria, if applicable | 4, 5, Fig S1 |
| Data sources/ measurement | 8* | For each variable of interest, give sources of data and details of methods of assessment (measurement). Describe comparability of assessment methods if there is more than one group | 4, 5 |
| Bias | 9 | Describe any efforts to address potential sources of bias | 6, 7 |
| Study size | 10 | Explain how the study size was arrived at | Fig 1 |
| Quantitative variables | 11 | Explain how quantitative variables were handled in the analyses. If applicable, describe which groupings were chosen and why | 6 |
| Statistical methods | 12 | (*a*) Describe all statistical methods, including those used to control for confounding | 6, 7 |
|  |  | (*b*) Describe any methods used to examine subgroups and interactions | 6 |
|  |  | (*c*) Explain how missing data were addressed | 6 |
|  |  | (*d*) If applicable, explain how loss to follow-up was addressed | 5 |
|  |  | (*e*) Describe any sensitivity analyses | 6, 7 |
| Results | | |  |
| Participants | 13* | (a) Report numbers of individuals at each stage of study—eg numbers potentially eligible, examined for eligibility, confirmed eligible, included in the study, completing follow-up, and analysed | 7, Fig 1 |
|  |  | (b) Give reasons for non-participation at each stage | 7, Fig 1 |
|  |  | (c) Consider use of a flow diagram | Fig 1 |
| Descriptive data | 14* | (a) Give characteristics of study participants (eg demographic, clinical, social) and information on exposures and potential confounders | 7, Tab 1 |
|  |  | (b) Indicate number of participants with missing data for each variable of interest | Tab 1 |
|  |  | (c) Summarise follow-up time (eg, average and total amount) | 7 |
| Outcome data | 15* | Report numbers of outcome events or summary measures over time | 7 |
| Main results | 16 | (*a*) Give unadjusted estimates and, if applicable, confounder-adjusted estimates and their precision (eg, 95% confidence interval). Make clear which confounders were adjusted for and why they were included | 7, 8, Tab 2, Fig 2, Fig 3, Fig S1 |
|  |  | (*b*) Report category boundaries when continuous variables were categorized | Tab 2 |
|  |  | (*c*) If relevant, consider translating estimates of relative risk into absolute risk for a meaningful time period | Not applicable |
| Other analyses | 17 | Report other analyses done—eg analyses of subgroups and interactions, and sensitivity analyses | 8 |
| Discussion | | | |
| Key results | 18 | Summarise key results with reference to study objectives | 8 |
| Limitations | 19 | Discuss limitations of the study, taking into account sources of potential bias or imprecision. Discuss both direction and magnitude of any potential bias | 11, 12 |
| Interpretation | 20 | Give a cautious overall interpretation of results considering objectives, limitations, multiplicity of analyses, results from similar studies, and other relevant evidence | 8-11 |
| Generalisability | 21 | Discuss the generalisability (external validity) of the study results | 12 |
| Other information | | | |
| Funding | 22 | Give the source of funding and the role of the funders for the present study and, if applicable, for the original study on which the present article is based | 13 |

**Figure S1**. Directed acyclic graph to guide covariate selection


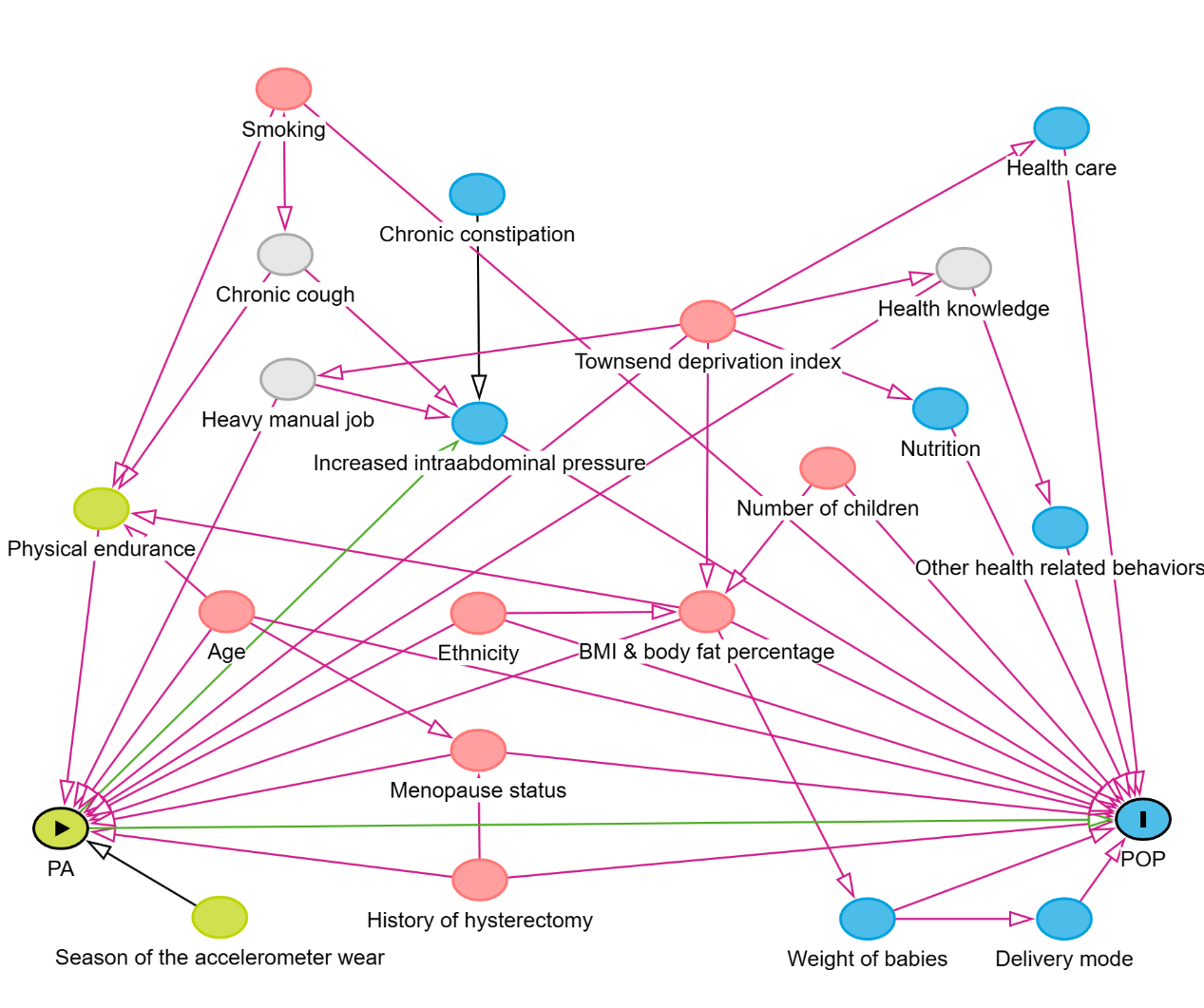


Green circle with a triangle inside: exposure; green circle without a triangle inside: ancestor of exposure; blue circle with a bar inside: outcome; blue circle without a bar inside: ancestor of outcome; pink circle: potential confounder that has been adjusted; grey circle: potential confounder that has not been adjusted due to lack of information; pink line: biasing path; green line and black line: causal path.

**Abbreviations**: BMI, body mass index; PA, physical activity; POP, pelvic organ prolapse

**Figure S2**. Stratified analyses of the association between LPA (per 1 h/day increment) and the risk of POP


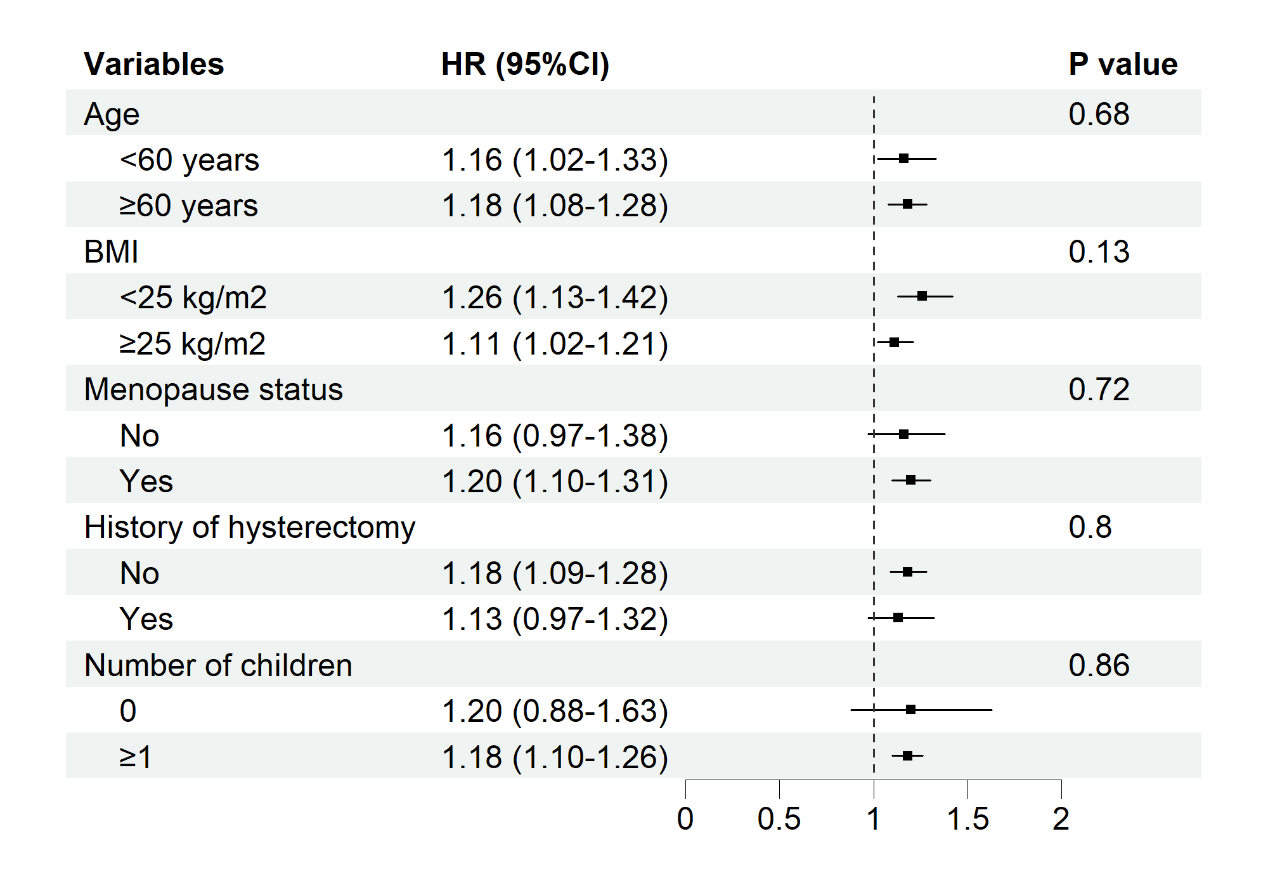
 **Abbreviations**: BMI, body mass index; CI, confidence interval; HR, hazard ratio; LPA, light-intensity physical activity; POP, pelvic organ prolapse

The model was adjusted for age when the accelerometry started (year, continuous), ethnicity (White, Black or Black British, Asian or Asian British, or mixed), Townsend deprivation index (continuous), BMI (kg/m^2^, continuous), body fat percentage (%, continuous), smoking status (never, previous, or current), menopause status (yes, no, not sure with a history of hysterectomy, or not sure with other reasons), history of hysterectomy (yes or no), and number of children (1, 2, or ≥2). The stratified factor was removed from the list of covariates in the adjustment.

**Figure S3**. Stratified analyses of the association between MPA (per 30min/day increment) and the risk of POP


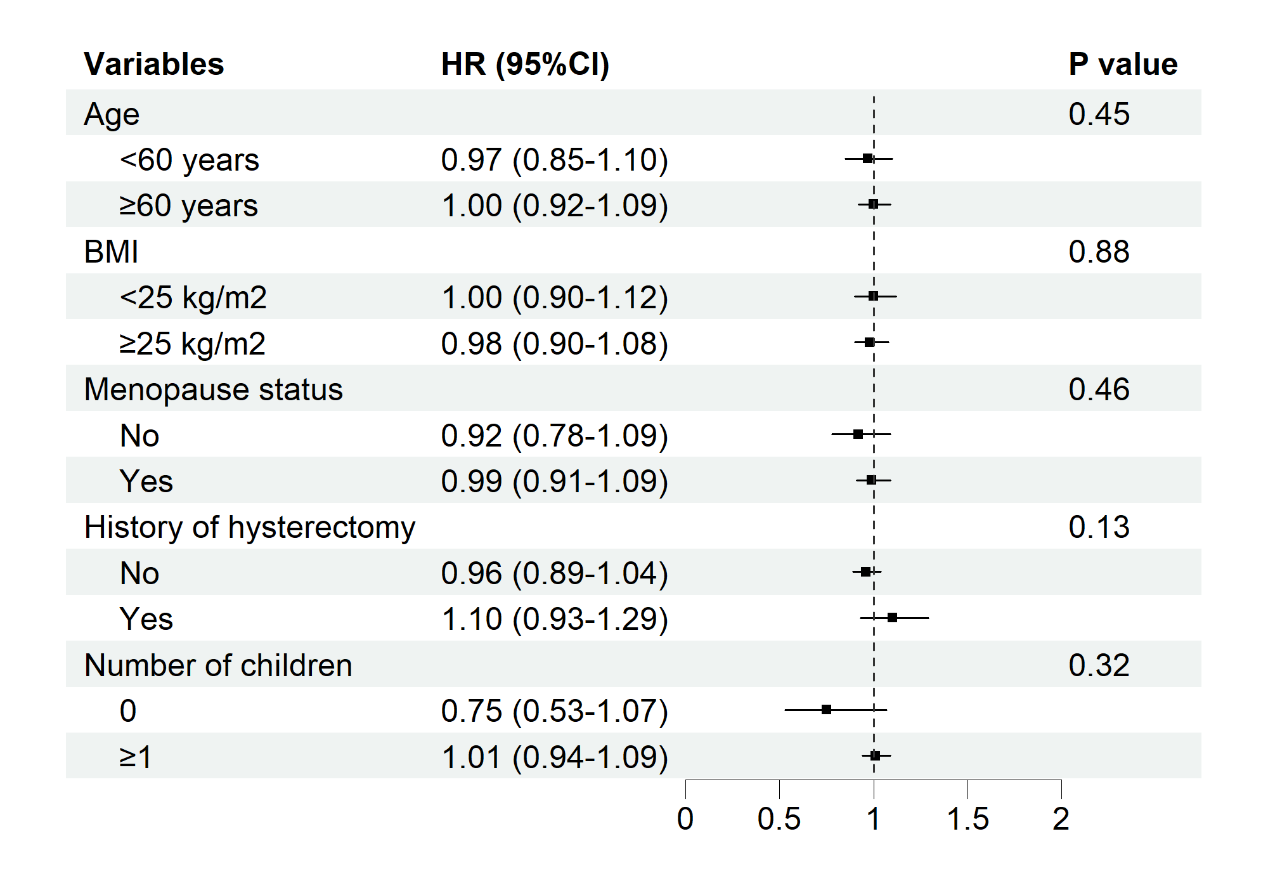


**Abbreviations**: BMI, body mass index; CI, confidence interval; HR, hazard ratio; MPA, moderate-intensity physical activity; POP, pelvic organ prolapse

The model was adjusted for age when the accelerometry started (year, continuous), ethnicity (White, Black or Black British, Asian or Asian British, or mixed), Townsend deprivation index (continuous), BMI (kg/m^2^, continuous), body fat percentage (%, continuous), smoking status (never, previous, or current), menopause status (yes, no, not sure with a history of hysterectomy, or not sure with other reasons), history of hysterectomy (yes or no), and number of children (0, 1, or ≥2). The stratified factor was removed from the list of covariates in the adjustment.

**Figure S4**. Stratified analyses of the association between VPA (per 15 min/day increment) and the risk of POP


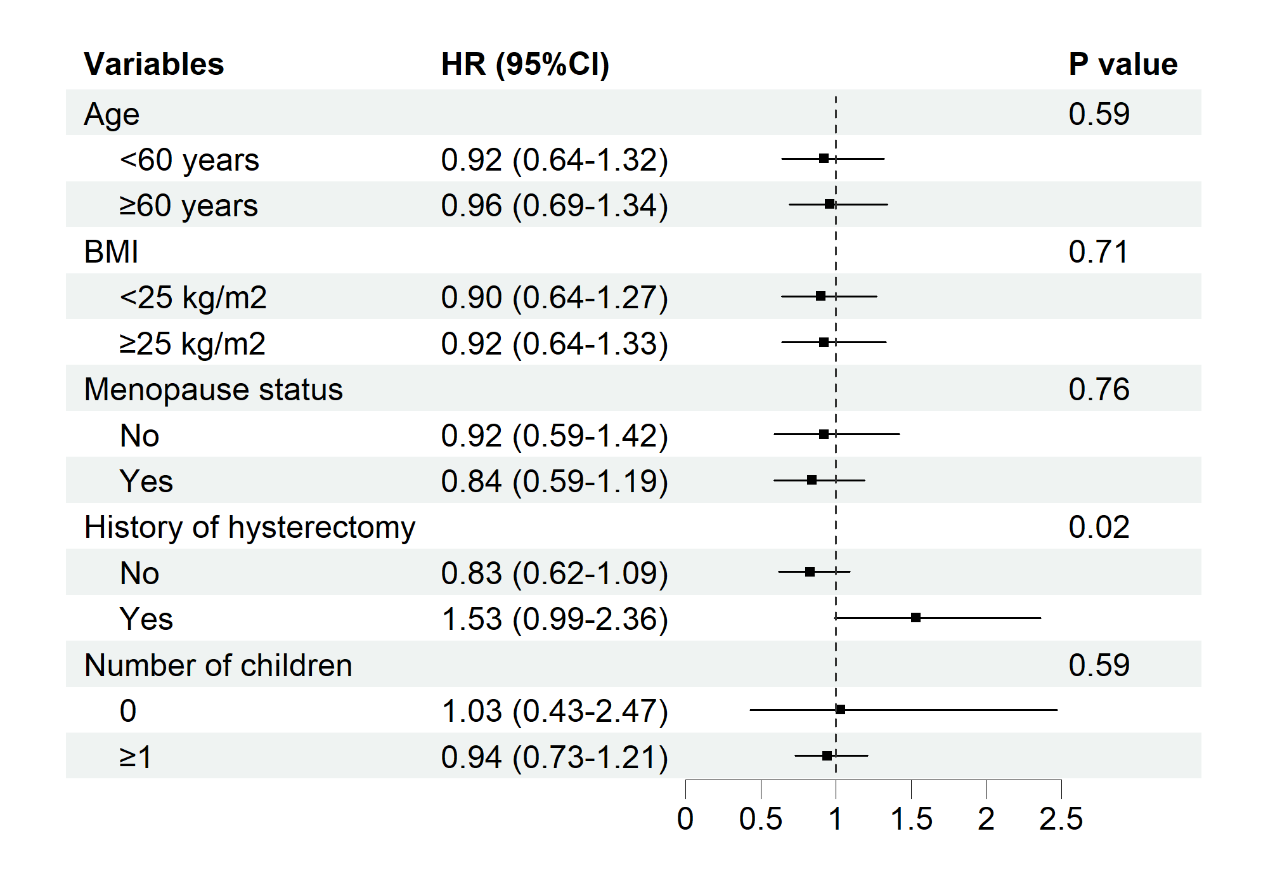


**Abbreviations**: BMI, body mass index; CI, confidence interval; HR, hazard ratio; VPA, vigorous-intensity physical activity; POP, pelvic organ prolapse

The model was adjusted for age when the accelerometry started (year, continuous), ethnicity (White, Black or Black British, Asian or Asian British, or mixed), Townsend deprivation index (continuous), BMI (kg/m^2^, continuous), body fat percentage (%, continuous), smoking status (never, previous, or current), menopause status (yes, no, not sure with a history of hysterectomy, or not sure with other reasons), history of hysterectomy (yes or no), and number of children (0, 1, or ≥2). The stratified factor was removed from the list of covariates in the adjustment.

**Figure S5**. Stratified analyses of the association between sedentary behavior (per 1h/day increment) and the risk of POP


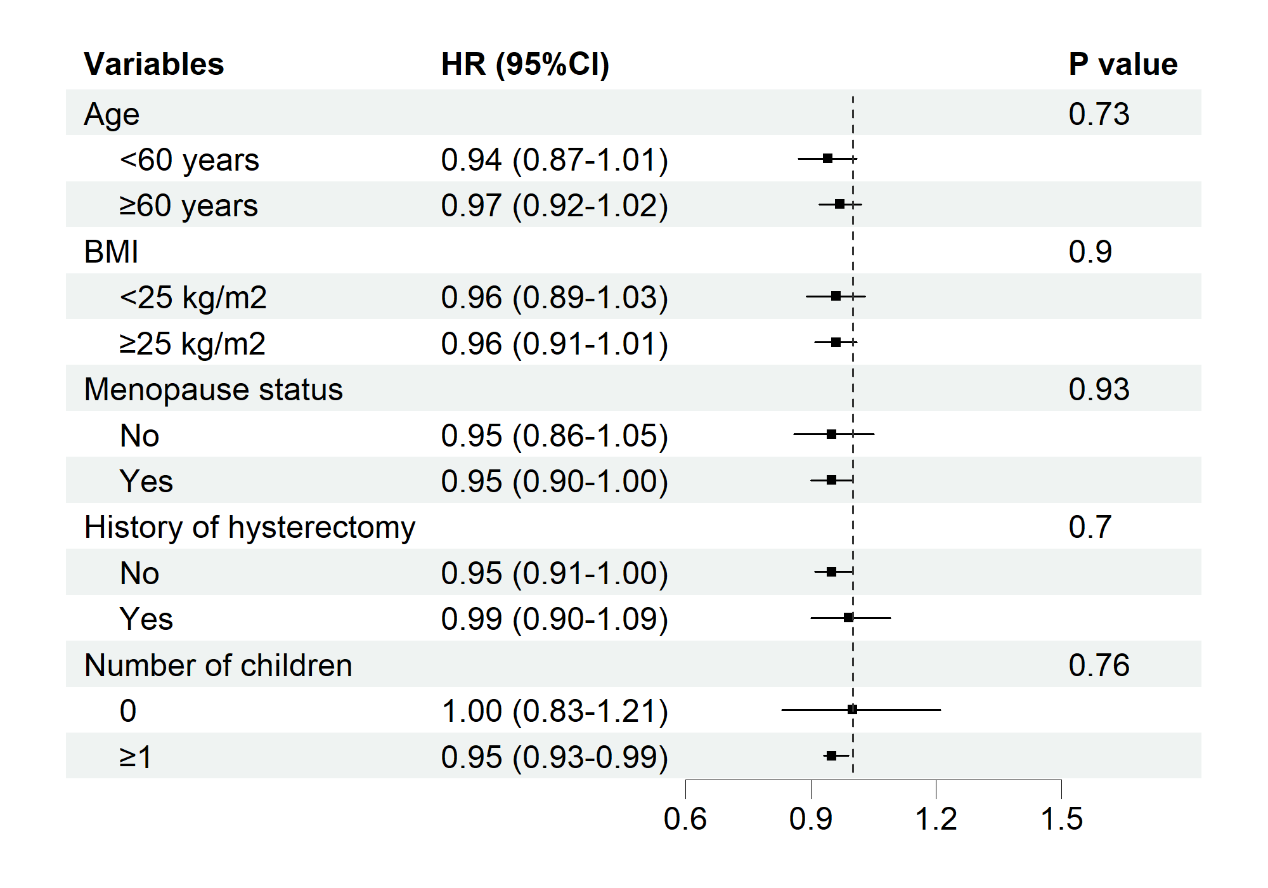


**Abbreviations**: BMI, body mass index; CI, confidence interval; HR, hazard ratio; POP, pelvic organ prolapse

The model was adjusted for age when the accelerometry started (year, continuous), ethnicity (White, Black or Black British, Asian or Asian British, or mixed), Townsend deprivation index (continuous), BMI (kg/m^2^, continuous), body fat percentage (%, continuous), smoking status (never, previous, or current), menopause status (yes, no, not sure with a history of hysterectomy, or not sure with other reasons), history of hysterectomy (yes or no), and number of children (0, 1, or ≥2). The stratified factor was removed from the list of covariates in the adjustment.

**Figure S6**. Dose-response association of PA and sedentary behavior with the risk of POP after excluding participants who were diagnosed with POP in the first two years of follow-up (N=47,190)


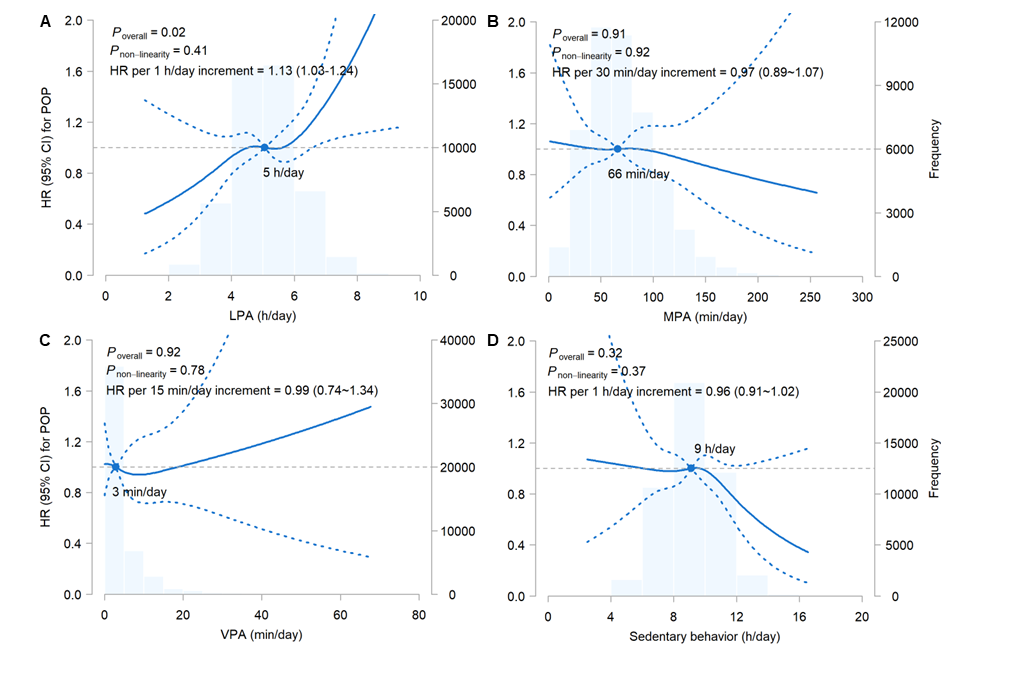


**Abbreviations**: CI, confidence interval; h, hour; HR, hazard ratio; LPA, light-intensity physical activity; min, minute; MPA, moderate-intensity physical activity; PA, physical activity; POP, pelvic organ prolapse; VPA, vigorous-intensity physical activity

The model was adjusted for age when the accelerometry started (year, continuous), ethnicity (White, Black or Black British, Asian or Asian British, or mixed), Townsend deprivation index (continuous), body mass index (kg/m^2^, continuous), body fat percentage (%, continuous), smoking status (never, previous, or current), menopause status (yes, no, not sure with a history of hysterectomy, or not sure with other reasons), history of hysterectomy (yes or no), and number of children (0, 1, or ≥2).

**Figure S7**. Dose-response association between PA and the risk of POP with mutual adjustment (N=47,674)


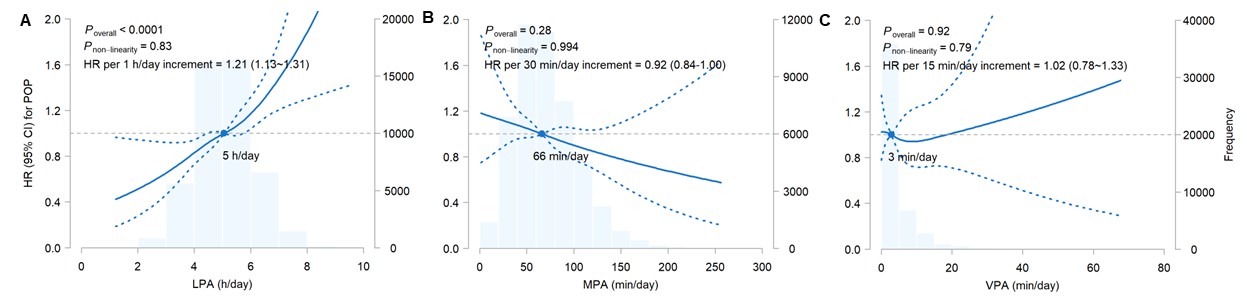


**Abbreviations**: CI, confidence interval; h, hour; HR, hazard ratio; LPA, light-intensity physical activity; min, minute; MPA, moderate-intensity physical activity; PA, physical activity; POP, pelvic organ prolapse; VPA, vigorous-intensity physical activity

The model was adjusted for age when the accelerometry started (year, continuous), ethnicity (White, Black or Black British, Asian or Asian British, or mixed), Townsend deprivation index (continuous), body mass index (kg/m^2^, continuous), body fat percentage (%, continuous), smoking status (never, previous, or current), menopause status (yes, no, not sure with a history of hysterectomy, or not sure with other reasons), history of hysterectomy (yes or no), and number of children (0, 1, or ≥2). Additionally, LPA (h/day, continuous), MPA (min/day, continuous), and VPA (min/day, continuous) were mutually adjusted.

**Figure S8**. Dose-response association of PA and sedentary behavior with the risk of POP in complete cases (N=46,283)


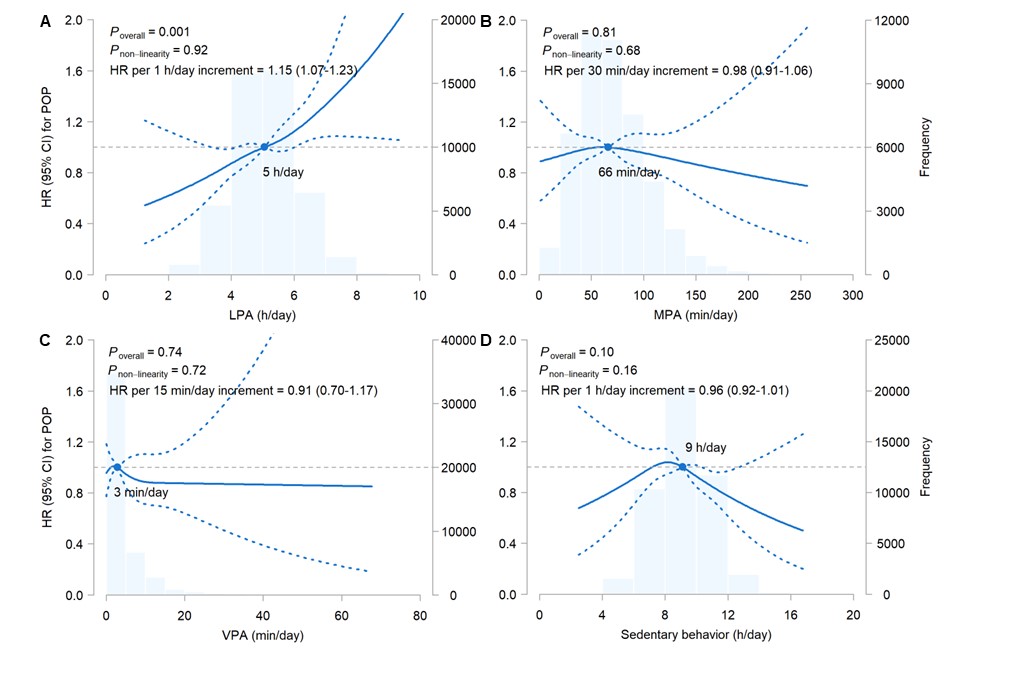


**Abbreviations**: CI, confidence interval; h, hour; HR, hazard ratio; LPA, light-intensity physical activity; min, minute; MPA, moderate-intensity physical activity; PA, physical activity; POP, pelvic organ prolapse; VPA, vigorous-intensity physical activity

The model was adjusted for age when the accelerometry started (year, continuous), ethnicity (White, Black or Black British, Asian or Asian British, or mixed), Townsend deprivation index (continuous), body mass index (kg/m^2^, continuous), body fat percentage (%, continuous), smoking status (never, previous, or current), menopause status (yes, no, not sure with a history of hysterectomy, or not sure with other reasons), history of hysterectomy (yes or no), and number of children (0, 1, or ≥2).

**Table S1**. Participant baseline characteristics by inclusion status

|  | **Included (N=47,674)** | **Excluded (N=225,646)** | **Overall (N=273,320)** |
| --- | --- | --- | --- |
| **Age (years)*** |  |  |  |
|  |  |  |  |
| Median (IQR) | 56.0 (13.0) | 58.0 (13.0) | 57.0 (13.0) |
| **Ethnicity, n (%)** |  |  |  |
| White | 45953 (96.4) | 211431 (93.7) | 257384 (94.2) |
| Mixed | 618 (1.3) | 3826 (1.7) | 4444 (1.6) |
| Asian or Asian British | 514 (1.1) | 5060 (2.2) | 5574 (2.0) |
| Black or Black British | 451 (0.9) | 4201 (1.9) | 4652 (1.7) |
| Missing | 138 (0.3) | 1128 (0.5) | 1266 (0.5) |
| **TDI** |  |  |  |
| Median (IQR) | -2.4 (3.7) | -2.1 (4.2) | -2.1 (4.1) |
| **BMI, kg/m^2^, n (%)** |  |  |  |
| <25 | 22334 (46.8) | 84417 (37.4) | 106751 (39.1) |
| 25~29.9 | 16446 (34.5) | 83403 (37.0) | 99849 (36.5) |
| ≥30 | 8506 (17.8) | 54906 (24.3) | 63412 (23.2) |
| Missing | 388 (0.8) | 2920 (1.3) | 3308 (1.2) |
| **Body fat percentage (%)** |  |  |  |
|  |  |  |  |
| Mean (SD) | 35.3 (6.8) | 36.8 (6.8) | 36.5 (6.8) |
| **Smoking, n (%)** |  |  |  |
| Never | 29066 (61.0) | 132953 (58.9) | 162019 (59.3) |
| Previous | 15648 (32.8) | 69779 (30.9) | 85427 (31.3) |
| Current | 2836 (5.9) | 21525 (9.5) | 24361 (8.9) |
| Missing | 124 (0.3) | 1389 (0.6) | 1513 (0.6) |
| **Number of children, n (%)** |  |  |  |
| 0 | 10805 (22.7) | 40283 (17.9) | 51088 (18.7) |
| 1 | 6187 (13.0) | 30272 (13.4) | 36459 (13.3) |
| ≥2 | 30644 (64.3) | 154299 (68.4) | 184943 (67.7) |
| Missing | 38 (0.1) | 792 (0.4) | 830 (0.3) |
| **Menopause status, n (%)** |  |  |  |
| No | 12631 (26.5) | 51409 (22.8) | 64040 (23.4) |
| Yes | 28027 (58.8) | 137351 (60.9) | 165378 (60.5) |
| Not sure | 6957 (14.6) | 35935 (15.9) | 42892 (15.7) |
| Missing | 59 (0.1) | 951 (0.4) | 1010 (0.4) |
| **History of hysterectomy, n (%)** |  |  |  |
| No | 40625 (85.2) | 180509 (80.0) | 221134 (80.9) |
| Yes | 6949 (14.6) | 43616 (19.3) | 50565 (18.5) |
| Missing | 100 (0.2) | 1521 (0.7) | 1621 (0.6) |

*****Age at baseline (2006-2010), instead of age at accelerometry (2013-2015) in table 1, was presented here because accelerometry was not conducted for most women in the excluded group, therefore age at accelerometry was not available for them.

**Abbreviations**: BMI, body mass index; IQR, interquartile range; SD, standard deviation; TDI, Townsend deprivation index.

**Table S2**. Joint association of LPA and MPA with the risk of POP

| **LPA (h/day)** | **MPA (min/day)** | **Cases/Person-years** | **Crude HR (95% CI)** | **Adjusted HR (95% CI)** |
| --- | --- | --- | --- | --- |
| **<4** | <30 | 21/14,401 | 1 (Reference) | 1 (Reference) |
|  | 30~<60 | 41/22,656 | 1.27 (0.75 to 2.16) | 1.61 (0.95 to 2.73) |
|  | **60~<90** | 20/10,535 | 1.34 (0.73 to 2.48) | **2.12 (1.14 to 3.94)** |
|  | 90~<120 | 2/2,826 | 0.50 (0.12 to 2.14) | 0.89 (0.21 to 3.82) |
|  | ≥120 | 2/754 | 1.89 (0.44 to 8.06) | 3.93 (0.92 to 16.84) |
| **4~<5** | **<30** | 37/11,116 | 2.31 (1.35 to 3.95) | **2.28 (1.34 to 3.91)** |
|  | **30~<60** | 115/51,431 | 1.57 (0.99 to 2.50) | **1.83 (1.15 to 2.92)** |
|  | **60~<90** | 73/42,114 | 1.22 (0.75 to 1.99) | **1.71 (1.05 to 2.81)** |
|  | 90~<120 | 26/16,662 | 1.10 (0.62 to 1.96) | 1.78 (0.99 to 3.19) |
|  | ≥120 | 4/5,837 | 0.49 (0.17 to 1.42) | 0.82 (0.28 to 2.41) |
| **5~<6** | **<30** | 14/4,178 | 2.33 (1.19 to 4.59) | **2.39 (1.22 to 4.71)** |
|  | **30~<60** | 83/37,067 | 1.58 (0.98 to 2.54) | **1.80 (1.11 to 2.91)** |
|  | **60~<90** | 105/46,787 | 1.58 (0.99 to 2.53) | **2.09 (1.30 to 3.37)** |
|  | **90~<120** | 45/25,819 | 1.23 (0.73 to 2.07) | **1.83 (1.08 to 3.10)** |
|  | ≥120 | 17/12,801 | 0.94 (0.50 to 1.78) | 1.59 (0.83 to 3.05) |
| **6~<7** | <30 | 3/763 | 2.74 (0.82 to 9.19) | 2.60 (0.78 to 8.73) |
|  | **30~<60** | 46/11,364 | 2.84 (1.70 to 4.76) | **3.26 (1.94 to 5.48)** |
|  | **60~<90** | 39/19,499 | 1.41 (0.83 to 2.40) | **1.81 (1.06 to 3.09)** |
|  | **90~<120** | 30/13,768 | 1.54 (0.88 to 2.69) | **2.22 (1.26 to 3.91)** |
|  | **≥120** | 19/8,613 | 1.57 (0.84 to 2.91) | **2.48 (1.32 to 4.67)** |
| **≥7** | <30 | 0/150 | NA* | NA* |
|  | **30~<60** | 12/2,113 | 3.98 (1.96 to 8.09) | **4.62 (2.27 to 9.43)** |
|  | **60~<90** | 14/4,460 | 2.20 (1.12 to 4.33) | **2.83 (1.43 to 5.60)** |
|  | 90~<120 | 6/3,432 | 1.24 (0.50 to 3.08) | 1.76 (0.71 to 4.39) |
|  | ≥120 | 5/2,933 | 1.21 (0.45 to 3.20) | 2.00 (0.75 to 5.36) |

**Abbreviations:** CI, confidence interval; h, hour; HR, hazard ratio; LPA, light-intensity physical activity; min, minute; MPA, moderate-intensity physical activity; NA, not available; POP, pelvic organ prolapse

The model was adjusted for age when the accelerometry started (year, continuous), ethnicity (White, Black or Black British, Asian or Asian British, or mixed), Townsend deprivation index (continuous), body mass index (kg/m^2^, continuous), body fat percentage (%, continuous), smoking status (never, previous, or current), menopause status (yes, no, not sure with a history of hysterectomy, or not sure with other reasons), history of hysterectomy (yes or no), and number of children (0, 1, or ≥2).

*There were not enough participants or cases in this category for analyses.

**Table S3**. Joint association of LPA and VPA with the risk of POP

| **LPA (h/day)** | **VPA (min/day)** | **Cases/Person-years** | **Crude HR (95% CI)** | **Adjusted HR (95% CI)** |
| --- | --- | --- | --- | --- |
| **<4** | 0 | 26/17,434 | 1 (Reference) | 1 (Reference) |
|  | **>0~34** | 52/25,984 | 1.59 (0.99 to 2.54) | **1.88 (1.17 to 3.01)** |
|  | 35~74 | 6/5,832 | 0.82 (0.34 to 1.99) | 1.19 (0.49 to 2.91) |
|  | 75~149 | 0/1,489 | NA* | NA* |
|  | **≥150** | 2/433 | 3.74 (0.89 to 15.75) | **7.47 (1.76 to 31.67)** |
| **4~<5** | **0** | 62/23,019 | 2.12 (1.34 to 3.35) | **2.01 (1.27 to 3.17)** |
|  | **>0~34** | 154/74,473 | 1.64 (1.08 to 2.48) | **1.84 (1.21 to 2.80)** |
|  | 35~74 | 33/24,400 | 1.08 (0.64 to 1.80) | 1.56 (0.93 to 2.64) |
|  | 75~149 | 6/4,261 | 1.12 (0.46 to 2.72) | 1.89 (0.77 to 4.62) |
|  | ≥150 | 0/1,006 | NA* | NA* |
| **5~<6** | **0** | 42/15,571 | 2.12 (1.30 to 3.46) | **2.03 (1.24 to 3.31)** |
|  | **>0~34** | 161/75,135 | 1.70 (1.12 to 2.57) | **1.93 (1.27 to 2.94)** |
|  | **35~74** | 49/29,826 | 1.31 (0.81 to 2.11) | **1.82 (1.12 to 2.96)** |
|  | **75~149** | 11/5,148 | 1.71 (0.84 to 3.46) | **2.80 (1.37 to 5.73)** |
|  | ≥150 | 1/972 | 0.82 (0.11 to 6.04) | 1.49 (0.20 to 11.02) |
| **6~<7** | **0** | 20/5,320 | 2.96 (1.65 to 5.30) | **2.84 (1.58 to 5.10)** |
|  | **>0~34** | 79/32,527 | 1.93 (1.24 to 3.00) | **2.20 (1.41 to 3.44)** |
|  | **35~74** | 32/13,614 | 1.87 (1.12 to 3.14) | **2.62 (1.55 to 4.44)** |
|  | **75~149** | 5/2,126 | 1.86 (0.72 to 4.86) | **2.97 (1.13 to 7.80)** |
|  | ≥150 | 1/420 | 1.86 (0.25 to 13.69) | 3.58 (0.48 to 26.61) |
| **≥7** | **0** | 8/1,294 | 4.87 (2.21 to 10.76) | **5.16 (2.33 to 11.42)** |
|  | **>0~34** | 23/8,173 | 2.23 (1.27 to 3.90) | **2.59 (1.47 to 4.56)** |
|  | 35~74 | 5/3,053 | 1.31 (0.50 to 3.42) | 1.92 (0.73 to 5.04) |
|  | 75~149 | 0/483 | NA* | NA* |
|  | **≥150** | 1/85 | 9.42 (1.28 to 69.41) | **21.49 (2.88 to 160.27)** |

**Abbreviations:** CI, confidence interval; h, hour; HR, hazard ratio; LPA, light-intensity physical activity; min, minute; NA, not available; VPA, vigorous-intensity physical activity; POP, pelvic organ prolapse

The model was adjusted for age when the accelerometry started (year, continuous), ethnicity (White, Black or Black British, Asian or Asian British, or mixed), Townsend deprivation index (continuous), body mass index (kg/m^2^, continuous), body fat percentage (%, continuous), smoking status (never, previous, or current), menopause status (yes, no, not sure with a history of hysterectomy, or not sure with other reasons), history of hysterectomy (yes or no), and number of children (0, 1, or ≥2).

*There were not enough participants or cases in this category for analyses.

**Table S4**. Joint association of MPA and VPA with the risk of POP

| **MPA (min/day)** | **VPA (min/day)** | **Cases/Person-years** | **Crude HR (95% CI)** | **Adjusted HR (95% CI)** |
| --- | --- | --- | --- | --- |
| <30 | 0 | 57/23,373 | 1 (Reference) | 1 (Reference) |
|  | >0~34 | 17/7,042 | 1.07 (0.63 to 1.85) | 1.13 (0.66 to 1.94) |
|  | 35~74 | 1/138 | 3.21 (0.45 to 23.21) | 6.40 (0.88 to 46.44) |
|  | 75~149 | 0/31 | NA* | NA* |
|  | ≥150 | 0/24 | NA* | NA* |
| 30~<60 | 0 | 86/32,321 | 1.19 (0.85 to 1.66) | 1.29 (0.92 to 1.81) |
|  | >0~34 | 199/83,628 | 1.07 (0.80 to 1.43) | 1.25 (0.93 to 1.68) |
|  | 35~74 | 11/7,178 | 0.69 (0.36 to 1.32) | 1.00 (0.52 to 1.93) |
|  | 75~149 | 1/1,233 | 0.37 (0.05 to 2.66) | 0.60 (0.08 to 4.32) |
|  | ≥150 | 0/270 | NA* | NA* |
| 60~<90 | 0 | 15/6,185 | 1.09 (0.61 to 1.92) | 1.34 (0.76 to 2.38) |
|  | >0~34 | 190/86,290 | 0.99 (0.74 to 1.33) | 1.29 (0.95 to 1.74) |
|  | 35~74 | 39/26,373 | 0.67 (0.44 to 1.00) | 1.02 (0.67 to 1.54) |
|  | 75~149 | 5/3,751 | 0.60 (0.24 to 1.50) | 1.05 (0.42 to 2.64) |
|  | ≥150 | 2/794 | 1.14 (0.28 to 4.69) | 2.34 (0.57 to 9.65) |
| 90~<120 | 0 | 0/662 | NA* | NA* |
|  | >0~34 | 54/31,265 | 0.78 (0.54 to 1.13) | 1.10 (0.75 to 1.62) |
|  | 35~74 | 47/25,851 | 0.82 (0.56 to 1.21) | 1.28 (0.86 to 1.91) |
|  | 75~149 | 8/3,914 | 0.92 (0.44 to 1.93) | 1.66 (0.78 to 3.53) |
|  | ≥150 | 0/817 | NA* | NA* |
| ≥120 | 0 | 0/97 | NA* | NA* |
|  | >0~34 | 9/8,067 | 0.50 (0.25 to 1.02) | 0.78 (0.38 to 1.58) |
|  | 35~74 | 27/17,185 | 0.71 (0.45 to 1.12) | 1.17 (0.73 to 1.88) |
|  | 75~149 | 8/4,579 | 0.79 (0.38 to 1.66) | 1.49 (0.70 to 3.17) |
|  | ≥150 | 3/1,011 | 1.33 (0.42 to 4.26) | 2.72 (0.84 to 8.80) |

**Abbreviations:** CI, confidence interval; h, hour; HR, hazard ratio; min, minute; MPA, moderate-intensity physical activity; NA, not available; VPA, vigorous-intensity physical activity; POP, pelvic organ prolapse

The model was adjusted for age when the accelerometry started (year, continuous), ethnicity (White, Black or Black British, Asian or Asian British, or mixed), Townsend deprivation index (continuous), body mass index (kg/m^2^, continuous), body fat percentage (%, continuous), smoking status (never, previous, or current), menopause status (yes, no, not sure with a history of hysterectomy, or not sure with other reasons), history of hysterectomy (yes or no), and number of children (0, 1, or ≥2).

*There were not enough participants or cases in these categories for analyses.

**Table S5**. Association of PA and sedentary behavior with the risk of POP after excluding participants who were diagnosed with POP in the first two years of follow-up (N=47,190)

| **Duration of Activity** | | **Cases/Person-year** | **Crude HR (95% CI)** | **Adjusted HR (95% CI)** |
| --- | --- | --- | --- | --- |
| LPA (h/day) | <4 | 49/51,079 | 1 (Reference) | 1 (Reference) |
|  | 4~<5 | 162/127,007 | 1.33 (0.97 to 1.83) | 1.33 (0.96 to 1.83) |
|  | 5~<6 | 154/126,482 | 1.27 (0.92 to 1.76) | 1.28 (0.92 to 1.77) |
|  | 6~<7 | 78/53,931 | 1.51 (1.06 to 2.16) | **1.53 (1.06 to 2.19)** |
|  | ≥ 7 | 23/13,072 | 1.84 (1.12 to 3.02) | **1.90 (1.15 to 3.13)** |
| MPA (min/day) | <30 | 46/30,506 | 1 (Reference) | 1 (Reference) |
|  | 30~<60 | 176/124,451 | 0.94 (0.68 to 1.31) | 1.06 (0.76 to 1.48) |
|  | 60~<90 | 153/123,267 | 0.83 (0.60 to 1.15) | 1.05 (0.75 to 1.48) |
|  | 90~<120 | 62/62,429 | 0.67 (0.45 to 0.97) | 0.92 (0.61 to 1.37) |
|  | ≥120 | 29/30,919 | 0.63 (0.40 to 1.00) | 0.94 (0.58 to 1.53) |
| VPA (min/day) | 0 | 94/62,489 | 1 (Reference) | 1 (Reference) |
|  | >0~<5 | 274/216,010 | 0.85 (0.67 to 1.07) | 0.98 (0.77 to 1.24) |
|  | 5~<15 | 81/76,663 | 0.71 (0.53 to 0.95) | 0.98 (0.71 to 1.34) |
|  | 15~<30 | 12/13,494 | 0.60 (0.33 to 1.09) | 0.95 (0.51 to 1.76) |
|  | ≥30 | 5/2,916 | 1.16 (0.47 to 2.84) | 2.08 (0.83 to 5.22) |
| Sedentary behavior (h/day) | <7 | 53/40,995 | 1 (Reference) | 1 (Reference) |
|  | 7~<8 | 71/54,948 | 1.00 (0.70 to 1.42) | 0.94 (0.66 to 1.34) |
|  | 8~<9 | 98/79,575 | 0.95 (0.68 to 1.33) | 0.89 (0.63 to 1.24) |
|  | 9~<10 | 111/83,759 | 1.02 (0.74 to 1.42) | 0.96 (0.69 to 1.33) |
|  | ≥10 | 133/112,295 | 0.91 (0.66 to 1.25) | 0.87 (0.63 to 1.21) |

**Abbreviations:** CI, confidence interval; h, hour; HR, hazard ratio; LPA, light-intensity physical activity; min, minute; MPA, moderate-intensity physical activity; PA, physical activity; POP, pelvic organ prolapse; VPA, vigorous-intensity physical activity

The model was adjusted for age when the accelerometry started (year, continuous), ethnicity (White, Black or Black British, Asian or Asian British, or mixed), Townsend deprivation index (continuous), body mass index (kg/m^2^, continuous), body fat percentage (%, continuous), smoking status (never, previous, or current), menopause status (yes, no, not sure with a history of hysterectomy, or not sure with other reasons), history of hysterectomy (yes or no), and number of children (0, 1, or ≥2).

**Table S6**. Association of PA and sedentary behavior with the risk of POP in competing risk models (N=47,674)

| **Duration of Activity** | | **Cases/Person-year** | **Crude HR (95% CI)** | **Adjusted HR (95% CI)** |
| --- | --- | --- | --- | --- |
| LPA (h/day) | <4 | 86/51,172 | 1 (Reference) | 1 (Reference) |
|  | 4~<5 | 255/127,160 | 1.21 (0.94 to 1.54) | 1.24 (0.97 to 1.59) |
|  | 5~<6 | 264/126,652 | 1.26 (0.98 to 1.60) | **1.33 (1.03 to 1.70)** |
|  | 6~<7 | 137/54,007 | 1.53 (1.17 to 2.00) | **1.63 (1.24 to 2.15)** |
|  | ≥ 7 | 37/13,088 | 1.70 (1.16 to 2.50) | **1.88 (1.27 to 2.77)** |
| MPA (min/day) | <30 | 75/30,608 | 1 (Reference) | 1 (Reference) |
|  | 30~<60 | 297/124,630 | 1.00 (0.78 to 1.29) | 1.18 (0.91 to 1.52) |
|  | 60~<90 | 251/123,393 | 0.86 (0.67 to 1.11) | 1.17 (0.89 to 1.54) |
|  | 90~<120 | 109/62,508 | 0.74 (0.55 to 0.99) | 1.11 (0.82 to 1.53) |
|  | ≥120 | 47/30,939 | 0.65 (0.45 to 0.93) | 1.07 (0.73 to 1.57) |
| VPA (min/day) | 0 | 158/62,639 | (Reference) | 1 (Reference) |
|  | >0~<5 | 469/216,292 | 0.88 (0.73 to 1.05) | 1.04 (0.86 to 1.25) |
|  | 5~<15 | 125/76,725 | 0.66 (0.54 to 0.84) | 0.97 (0.75 to 1.25) |
|  | 15~<30 | 22/13,508 | 0.67 (0.44 to 1.04) | 1.12 (0.70 to 1.77) |
|  | ≥30 | 5/2,916 | 0.70 (0.29 to 1.71) | 1.36 (0.56 to 3.34) |
| Sedentary behavior (h/day) | <7 | 82/41,034 | 1 (Reference) | 1 (Reference) |
|  | 7~<8 | 125/55,033 | 1.13 (0.86 to 1.49) | 1.06 (0.80 to 1.39) |
|  | 8~<9 | 174/79,676 | 1.09 (0.84 to 1.42) | 1.01 (0.77 to 1.31) |
|  | 9~<10 | 176/83,861 | 1.05 (0.80 to 1.36) | 0.96 (0.74 to 1.24) |
|  | ≥10 | 222/112,475 | 0.98 (0.76 to 1.26) | 0.89 (0.69 to 1.15) |

**Abbreviations:** CI, confidence interval; h, hour; HR, hazard ratio; LPA, light-intensity physical activity; min, minute; MPA, moderate-intensity physical activity; PA, physical activity; POP, pelvic organ prolapse; VPA, vigorous-intensity physical activity

The model was adjusted for age when the accelerometry started (year, continuous), ethnicity (White, Black or Black British, Asian or Asian British, or mixed), Townsend deprivation index (continuous), body mass index (kg/m^2^, continuous), body fat percentage (%, continuous), smoking status (never, previous, or current), menopause status (yes, no, not sure with a history of hysterectomy, or not sure with other reasons), history of hysterectomy (yes or no), and number of children (0, 1, or ≥2).

**Table S7**. Association between PA and the risk of POP with mutual adjustment (N=47,674)

| **Duration of Activity** | | **Cases/Person-year** | **Crude HR (95% CI)** | **Adjusted HR (95% CI)** |
| --- | --- | --- | --- | --- |
| LPA (h/day) | <4 | 86/51,172 | 1 (Reference) | 1 (Reference) |
|  | 4~<5 | 255/127,160 | 1.20 (0.94 to 1.53) | **1.28 (1.00 to 1.64)** |
|  | 5~<6 | 264/126,652 | 1.25 (0.98 to 1.59) | **1.40 (1.08 to 1.80)** |
|  | 6~<7 | 137/54,007 | 1.52 (1.16 to 1.99) | **1.76 (1.31 to 2.35)** |
|  | ≥ 7 | 37/13,088 | 1.69 (1.15 to 2.48) | **2.05 (1.37 to 3.07)** |
| MPA (min/day) | <30 | 75/30,608 | 1 (Reference) | 1 (Reference) |
|  | 30~<60 | 297/124,630 | 0.99 (0.76 to 1.27) | 1.00 (0.77 to 1.31) |
|  | 60~<90 | 251/123,393 | 0.84 (0.65 to 1.09) | 0.92 (0.69 to 1.23) |
|  | 90~<120 | 109/62,508 | 0.73 (0.54 to 0.97) | 0.84 (0.60 to 1.18) |
|  | ≥120 | 47/30,939 | 0.63 (0.44 to 0.91) | 0.77 (0.50 to 1.19) |
| VPA (min/day) | 0 | 158/62,639 | 1 (Reference) | 1 (Reference) |
|  | >0~<5 | 469/216,292 | 0.87 (0.72 to 1.04) | 1.03 (0.84 to 1.26) |
|  | 5~<15 | 125/76,725 | 0.66 (0.52 to 0.83) | 1.02 (0.76 to 1.37) |
|  | 15~<30 | 22/13,508 | 0.66 (0.42 to 1.02) | 1.24 (0.76 to 2.03) |
|  | ≥30 | 5/2,916 | 0.69 (0.28 to 1.69) | 1.56 (0.62 to 3.92) |

**Abbreviations:** CI, confidence interval; h, hour; HR, hazard ratio; LPA, light-intensity physical activity; min, minute; MPA, moderate-intensity physical activity; PA, physical activity; POP, pelvic organ prolapse; VPA, vigorous-intensity physical activity

The model was adjusted for age when the accelerometry started (year, continuous), ethnicity (White, Black or Black British, Asian or Asian British, or mixed), Townsend deprivation index (continuous), body mass index (kg/m^2^, continuous), body fat percentage (%, continuous), smoking status (never, previous, or current), menopause status (yes, no, not sure with a history of hysterectomy, or not sure with other reasons), history of hysterectomy (yes or no), and number of children (0, 1, or ≥2). Additionally, LPA (h/day, continuous), MPA (min/day, continuous), and VPA (min/day, continuous) were mutually adjusted.

**Table S8**. Association of PA and sedentary behavior with the risk of POP in complete cases (N=46,283)

| **Duration of Activity** | | **Cases/Person-year** | **Crude HR (95% CI)** | **Adjusted HR (95% CI)** |
| --- | --- | --- | --- | --- |
| LPA (h/day) | <4 | 86/49,179 | 1 (Reference) | 1 (Reference) |
|  | 4~<5 | 250/123,673 | 1.16 (0.91 to 1.48) | 1.20 (0.94 to 1.54) |
|  | 5~<6 | 257/123,169 | 1.20 (0.94 to 1.53) | 1.27 (0.99 to 1.62) |
|  | 6~<7 | 132/52,689 | 1.44 (1.10 to 1.89) | **1.54 (1.17 to 2.03)** |
|  | ≥ 7 | 33/12,598 | 1.51 (1.01 to 2.25) | **1.65 (1.10 to 2.48)** |
| MPA (min/day) | <30 | 74/29,071 | 1 (Reference) | 1 (Reference) |
|  | 30~<60 | 289/120,837 | 0.95 (0.74 to 1.23) | 1.12 (0.86 to 1.45) |
|  | 60~<90 | 243/120,427 | 0.81 (0.62 to 1.05) | 1.10 (0.84 to 1.44) |
|  | 90~<120 | 106/60,898 | 0.70 (0.52 to 0.94) | 1.06 (0.77 to 1.45) |
|  | ≥120 | 46/30,074 | 0.61 (0.42 to 0.89) | 1.02 (0.69 to 1.51) |
| VPA (min/day) | 0 | 151/59,979 | 1 (Reference) | 1 (Reference) |
|  | >0~<5 | 458/210,430 | 0.87 (0.73 to 1.05) | 1.04 (0.86 to 1.26) |
|  | 5~<15 | 124/74,849 | 0.67 (0.53 to 0.85) | 0.99 (0.76 to 1.27) |
|  | 15~<30 | 21/13,272 | 0.64 (0.40 to 1.01) | 1.09 (0.68 to 1.74) |
|  | ≥30 | 4/2,778 | 0.58 (0.22 to 1.58) | 1.16 (0.42 to 3.16) |
| Sedentary behavior (h/day) | <7 | 76/39,916 | 1 (Reference) | 1 (Reference) |
|  | 7~<8 | 121/53,643 | 1.18 (0.89 to 1.57) | 1.10 (0.83 to 1.47) |
|  | 8~<9 | 172/77,541 | 1.16 (0.89 to 1.52) | 1.07 (0.81 to 1.40) |
|  | 9~<10 | 171/81,579 | 1.10 (0.84 to 1.44) | 1.00 (0.76 to 1.31) |
|  | ≥10 | 218/108,630 | 1.05 (0.81 to 1.36) | 0.95 (0.73 to 1.24) |

**Abbreviations:** CI, confidence interval; h, hour; HR, hazard ratio; LPA, light-intensity physical activity; min, minute; MPA, moderate-intensity physical activity; PA, physical activity; POP, pelvic organ prolapse; VPA, vigorous-intensity physical activity

The model was adjusted for age when the accelerometry started (year, continuous), ethnicity (White, Black or Black British, Asian or Asian British, or mixed), Townsend deprivation index (continuous), body mass index (kg/m^2^, continuous), body fat percentage (%, continuous), smoking status (never, previous, or current), menopause status (yes, no, not sure with a history of hysterectomy, or not sure with other reasons), history of hysterectomy (yes or no), and number of children (0, 1, or ≥2).
